# Genome-Wide Identification and Characterization of CCT Gene Family from Microalgae to Legumes

**DOI:** 10.3390/genes15070941

**Published:** 2024-07-18

**Authors:** Yi Xu, Huiying Yao, Yanhong Lan, Yu Cao, Qingrui Xu, Hui Xu, Dairong Qiao, Yi Cao

**Affiliations:** Microbiology and Metabolic Engineering Key Laboratory of Sichuan Province, Key Laboratory of Bio-Resources and Eco-Environment of Ministry of Education, College of Life Sciences, Sichuan University, Chengdu 610065, Chinacaoyu1984@163.com (Y.C.);

**Keywords:** CCT, gene structure, phylogenetic analysis, whole-genome duplication, collinearity analysis, expression pattern

## Abstract

The CCT (CO, COL and TOC1) gene family has been elucidated to be involved in the functional differentiation of the products in various plant species, but their specific mechanisms are poorly understood. In the present investigation, we conducted a genome-wide identification and phylogenetic analysis of CCT genes from microalgae to legumes. A total of 700 non-redundant members of the CCT gene family from 30 species were identified through a homology search. Phylogenetic clustering with *Arabidopsis* and domain conservation analysis categorized the CCT genes into three families. Multiple sequence alignment showed that the CCT domain contains important amino acid residues, and each CCT protein contains 24 conserved motifs, as demonstrated by the motif analysis. Whole-genome/segment duplication, as well as tandem duplication, are considered to be the driving forces in the evolutionary trajectory of plant species. This comprehensive investigation into the proliferation of the CCT gene family unveils the evolutionary dynamics whereby WGD/segment duplication is the predominant mechanism contributing to the expansion of the CCT genes. Meanwhile, the examination of the gene expression patterns revealed that the expression patterns of CCT genes vary in different tissues and at different developmental stages of plants, with high expression in leaves, which is consistent with the molecular regulation of flowering in photosynthesis by CCT. Based on the protein–protein interaction analysis of CCT genes in model plants, we propose that the CCT gene family synergistically regulates plant development and flowering with light-signaling factors (PHYs and PIFs) and MYB family transcription factors. Understanding the CCT gene family’s molecular evolution enables targeted gene manipulation for enhanced plant traits, including optimized flowering and stress resistance.

## 1. Introduction

The CCT (CONSTANS, CO-LIKE and TOC1) gene family was elucidated to participate in various plant life activities, such as photoperiodic flowering, light signaling, and circadian rhythm regulation [1,2,3]. In photosynthetic plants, the CCT superfamily is divided into three sub-families based on their motifs: COL (CONSTANS-like), CMF (CCT motif family), and PRR [4,5,6]. The COL sub-family genes contain a CCT domain and B-boxes, while the CMF sub-family genes only contain one CCT domain. The PRRs share a conserved domain, the receiver-like domain (RLD), along with a CCT domain [7]. Based on structural characteristics, the PRR in terrestrial plants is divided into three branches, including PRR1, PRR7/3, and PRR5/9 [8], which play important roles in the biological clock system of *Arabidopsis*. The transcription and translation order is PRR9 → PRR7 → PRR5 → PRR3 → PRR1, as confirmed by Matsushika et al. [9]. At present, the molecular functions of the PRR family members are not clear yet, but their biological functions are clearly related to circadian rhythms.

Nowadays, the CCT genes of the three sub-families have been widely elucidated in various model plants, such as *Arabidopsis* [7], rice (*Oryza sativa*), maize (*Zea mays*) and wheat (*Triticum aestivum*) [10,11,12]. CO (CONSTANS), a circadian clock regulatory gene belonging to the COL sub-family, is the first cloned CCT gene that controls the flowering time in *Arabidopsis* [7]. Ghd7 belongs to the CMF sub-family, and it functions upstream of Ehd1 and Hd3a in the photoperiod flowering pathway [13]. The genes of the COL sub-family have regulatory effects on flowering. Specifically, OsCO3 can downregulate the expression levels of FT-like genes, leading to early flowering under SD conditions [14]. OsCOL4, a constitutive flowering repressor, acts upstream of Ehd1 and downstream of OsphyB [15]. OsGhd2 is a pleiotropic gene that participates in regulating various physiological processes in rice through interactions with different proteins, including the grain number, heading date and plant height. It also endows rice with sensitivity to drought by accelerating drought-induced leaf senescence [16]. The PRR sub-family genes are functionally more conserved than the COL and CMF genes. As an important component of the circadian clock, AtPRR1, also known as TOC1, interacts with CCA1 (CIRCADIAN CLOCK ASSOCIATED 1) and LHY (LATE ELONGATED HYPOCOTYL) to form a feedback loop of the circadian clock that regulates plant flowering [17,18]. Although the CCT family has been functionally identified, there is a lack of more comprehensive analysis of the plant CCT gene family in terms of the phylogenetic, structural and functional evolution.

The gene structure, evolution and functional differentiation of the CCT gene family have not been studied systematically. With the latest advances in sequencing technologies, the number of sequenced plant genomes has sharply increased in recent years, providing us with the possibility of studying the CCT gene family from a broader perspective. Here, CCT proteins were identified from 30 plant species with different taxonomic relationships (from microalgae to legumes) at the whole-genome level, and the structures of CCT gene family members were investigated. Meanwhile, the evolutionary relationships and expression patterns of different members of the gene family were also explored. Furthermore, using functional diversity and positive selection analysis, we explored the evolution patterns of the gene structure and function in the CCT gene family. In brief, the present results will provide valuable information for further investigation of the functions and regulatory mechanisms of the CCT gene family in plants. Not only can it deepen our understanding of the role of the CCT gene family in plant physiology and development but also provide potential molecular targets for crop improvement and agricultural production.

## 2. Materials and Methods

### 2.1. Data Sources and Identification of CCT Proteins

Genome and annotation data of 30 representative plant species were obtained from the Plant JGI Database Phytozome v13 (accessed on 6 October 2022) [19] and National Center for Biotechnology Information Genome database (accessed on 22 December 2022). To identify putative CCT genes in the plant species, we created a local database of the CCT protein sequences from *Arabidopsis thaliana* [20] (GeneID: PRR1, AT5G61380; PRR3, AT5G60100; PRR5, AT5G24470; PRR7, AT5G02810; PRR9, AT2G46790; COL3, AT2G24790, COL1, AT1G68520; CMF1, AT1G05290, CMF5, AT2G33350). We used BLASTP to identify candidate CCTs in the local database. The e-value of 10^−5^ and scores > 200-bit were kept as the cut-off to identify candidate CCT genes. Finally, the BLASTP output was presented in tabular form [21]. The Conserved Domain Database (CDD) (accessed on 8 January 2020) [22], Pfam [23], and SMART (accessed on 26 October 2020) [24] were used to confirm the CCT proteins containing conserved domains of the CCT family. The locations of the CCT genes for all the species were extracted from the corresponding GFF files using an in-house Perl script.

### 2.2. Phylogenetic Analysis

The protein sequences of the 700 candidate CCT genes were used for the phylogenetic analysis, and the gene information is listed in Table S1. Multiple sequence alignments of the selected CCT sequences were performed via ClustalW and ClustalX with the default parameters [25]. The neighbor-joining (NJ) method was used to construct unrooted phylogenetic trees of the CCT proteins using MEGA (v7.0.26) [26]. FigTree (v1.4.3) was used to visualize the phylogenetic trees.

### 2.3. Motif Composition and Gene Structure Analysis

The protein sequences of all the genes were imported to the MEME tool (http://meme-suite.org/tools/meme (accessed on 17 December 2022)) [27] to analyze the conserved motifs, with a maximum of twenty-four most-conserved motifs per sequence. The resulting protein structures were visualized using TBtools [28].

### 2.4. Gene Expression Analysis

The expression data of the plant CCT family genes in different tissues and under different abiotic stresses were downloaded from Bio-Analytic Resource for Plant Biology (BARPB, accessed on 7 January 2022). The expression levels of the selected plant CCT genes were processed and displayed using TBtools [28].

### 2.5. Chromosome Location, Synteny Analysis, and Selective Force Analysis

Duplicated gene pairs among the selected plants were identified using the MCScanX [29], with an e-value of 1 × 10^−5^ in the BlastP search [21]. TBtools was used to visualize the syntenic relationships between the identified CCT genes. For the selective force analysis of the duplicated gene pairs, TBtools was also used to calculate the nonsynonymous/synonymous substitution (Ka/Ks) rate during the evolutionary process (Ka/Ks < 1, purifying; or Ka/Ks > 1, positive) [28].

### 2.6. Protein–Protein Interaction Networks Analysis

Based on the orthoVenn2 analysis and InParanoid v6 software [30] and STRING database using protein sequences in *A. thaliana*, *O. sativa* and *Glycine max*, an e-value of 10^−5^ and an inflation value > 1.5 scores were kept as the cut-off to identify putative CCT homologous proteins. The obtained homologous interaction protein pairs were edited and displayed using Cytoscape software (v3.10.2) [31].

## 3. Results

### 3.1. Comprehensive Identification and Distribution of CCT Genes in Plants

To identify the CCT protein families in the selected plants, the protein sequences of *A. thaliana* CCT proteins [20] were used as a reference to perform BLASTP. As a result, a total of 700 CCT candidates and their gene ID were obtained from 30 plant species (Table 1 and Table S1), including 11 eudicots, 7 microalgae (5 bacillariophyta and 2 chlorophyta), 2 phaeophyta, 5 monocots, 2 mosses, 1 fern (*Ceratopteris richardii*), 1 liverwort (*Marchantia polymorpha*) and 1 basal angiosperm (*Amborella trichopoda*). Furthermore, the 700 CCT candidates were classified into three sub-families based on their additional domains. The PRR sub-family genes were present in 30 selected plants, while 21 plant species contained COL sub-family genes, except for algae (Bacillariophyta, Chlorophyta, and Phaeophyta). The CMF sub-family genes were only found in 11 species of eudicots. The CCT copy number varied between 2 and 117, with the PRR sub-family and CMF sub-family having the highest and lowest CCT gene numbers, respectively. Among all the surveyed species, *G. hirsutum* had 117 CCT genes, while the algae genomes harbored the lowest CCT copies, whether microalgae or macroalgae. The PRR sub-family members were present in all the species analyzed, while the CMF sub-family members and COL sub-family genes were only found in the eudicots or land plants, respectively (Table 1). Through the PhenGram online server, the genomic chromosomal distribution of the identified CCT genes was mapped to the corresponding chromosomes based on their chromosomal positions in the plant genome. The results above showed that the CCT genes were distributed unevenly on the chromosomes or scaffolds in different species (Table S1).

### 3.2. Gene Structure, Conserved Domain and Phylogenetic Relationship of CCT Genes

As analyzed by the SMART program, the 700 CCT proteins have a characteristic central CCT domain (Figure S1). Multiple sequence alignments were conducted using the CCT domain protein sequences extracted from representative model plants (*A. thaliana*, *G. max*, and *O. sativa*). The CCT proteins were grouped into three sub-families, namely PRR, COL and CMF. The first sub-cluster is the PRR sub-family, which includes 408 CCT genes, all of which contain both CCT and REC domains. From microalgae to higher plants, the CCT gene family has undergone significant expansion in both numbers and types. The second sub-cluster includes 189 CCT genes that contain both CCT and BBOX domains, belonging to the COL sub-family. The third sub-cluster includes 103 CCT genes, belonging to the CMF sub-family, which only contain the CCT domain. In order to infer the structural variations and possible functional divergences of the CCT gene, MEME was used to analyze its coding sequences. The analysis results showed 24 conserved motifs, and the types and quantities of these conserved motifs were highly variable among the sub-families. Compared with CMF and COL, the PRR sub-family contained the highest number of motifs (Table S2). Motif 2 and motif 6 were widely distributed in all the CCT proteins and were located in the conserved CCT domain (Figures S2 and S3). Some motifs existed only in specific sub-families, such as motif 1, which only exists in the PRR sub-family.

**Figure 1:**
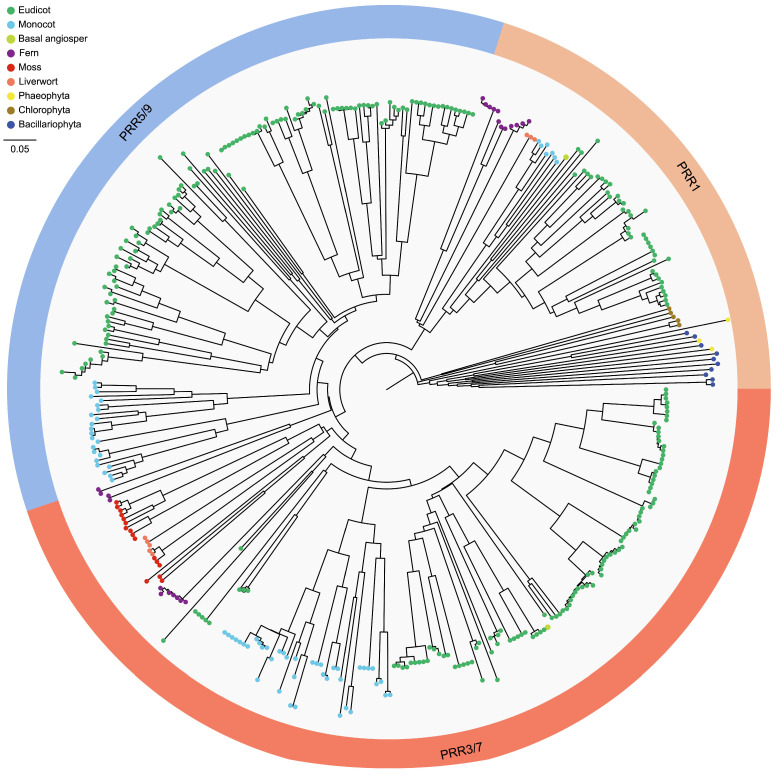
Phylogenetic analysis of the PRR gene sub-family from 30 species using MEGA7 software (v7.0.26) with the neighbor-joining method.

**Figure 2:**
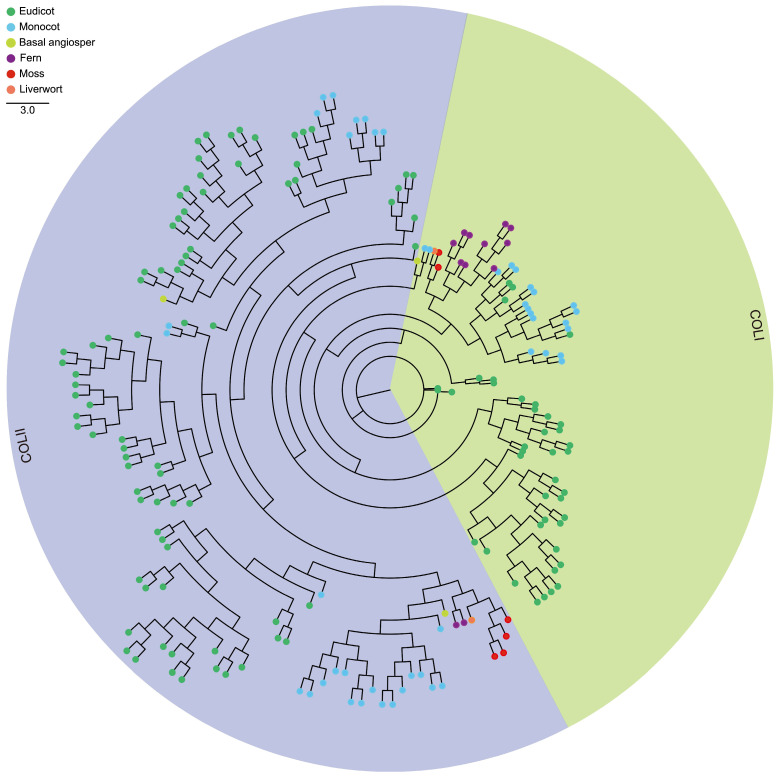
Phylogenetic evolutionary tree of the COL sub-family members from 21 species using MEGA7 software (v7.0.26) with the neighbor-joining method.

**Figure 3:**
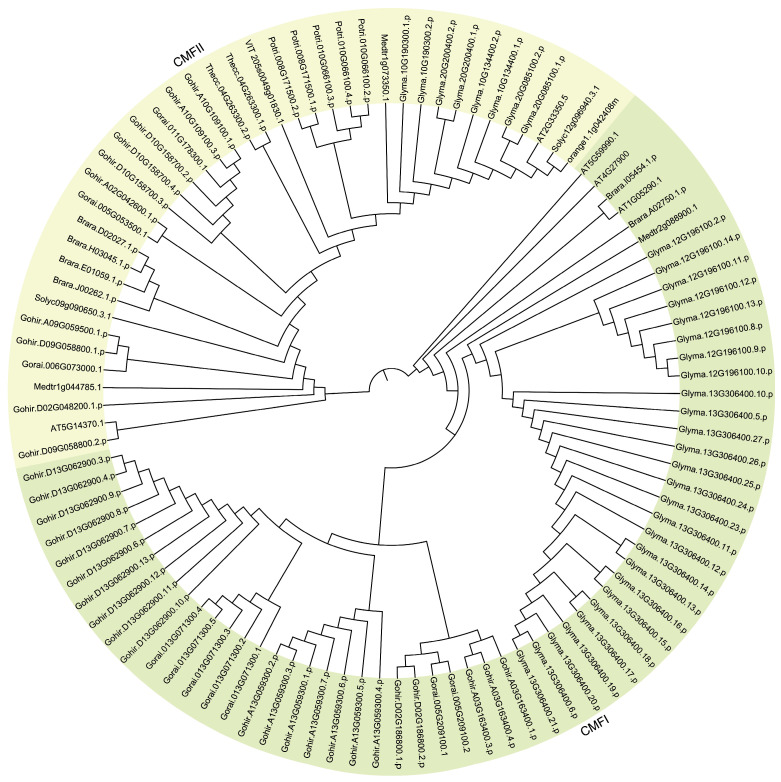
Phylogenetic evolutionary tree of the CMF sub-family members from 11 eudicot species using MEGA7 software (v7.0.26) with the neighbor-joining method.

To ensure the reliability of the analysis, neighbor-joining (NJ) was further used to perform multiple protein sequences alignment on the 700 CCT genes mentioned above, and an independent unrooted phylogenetic tree was constructed for each sub-family (Figure 1, Figure 2 and Figure 3). Obviously, the phylogenetic clustering of the CCT genes was consistent with the distribution of their conserved domains. The PRR clade was divided into three sub-clades, PRR1, PRR7/3, and PRR5/9. All the PRR genes of the microalgae and macroalgae were clustered in the PRR1 clade. The COL sequences formed two distinct clades, and the CMF sequences also formed two completely different clades. In general, the phylogeny of the CCT genes is largely related to the evolutionary relationships between species, and the genes of phylogenetically related species tend to gather in the tree.

### 3.3. Expansion Pattern of CCT Genes and Collinearity Analysis

**Figure 4:**
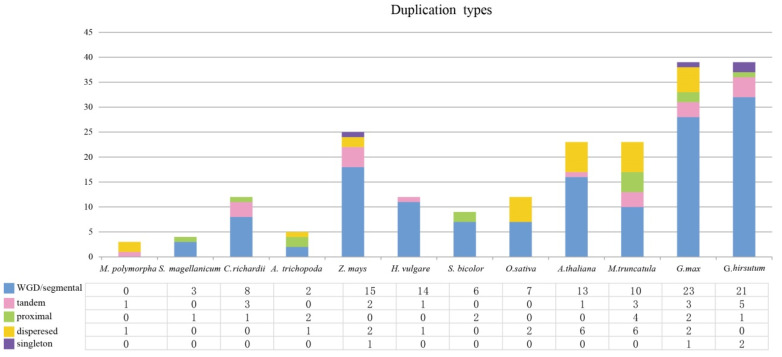
CCT gene family expansion from liverwort to angiosperms. Duplication types were analyzed in *M. polymorpha*, *S. magellanicum*, *C. richardii*, *A. trichopoda*, *S. bicolor*, *Z. mays*, *H. vulgare*, *O. sativa*, *A. thaliana*, *M. truncatula*, *G. max* and *G. hirsutum*.

In order to identify the expansion patterns of CCTs in land plants, we investigated the duplication types of the CCT genes in angiosperms, liverworts, ferns, and mosses. Overall, WGD, segmental, and dispersed duplications contributed the most to the expansion of the CCT genes. Specifically, the analysis revealed that expansion patterns in *Z. mays*, *G. max*, and *G. hirsutum* mainly arose from WGD/segmental duplication (Figure 4).

**Figure 5:**
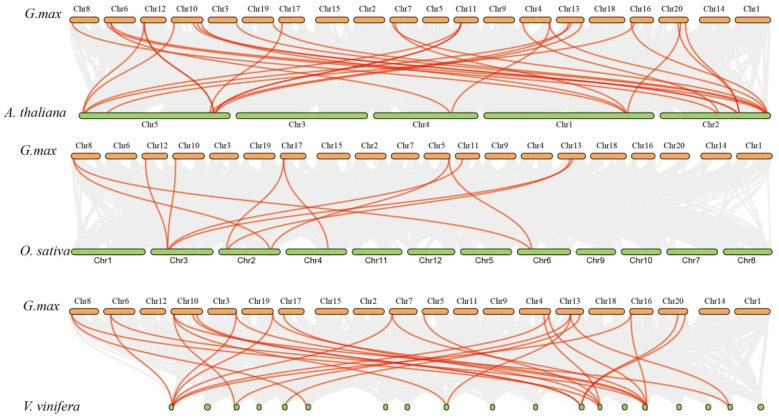
Interspecific collinearity analysis of CCT genes between *G. max*, *O. sativa*, *A. thaliana*, and *V. vinifera*. The gray lines in the background indicate collinearity between *G. max* and the 3 representative plant genomes, while the red lines show the syntenic CCT gene pairs.

To further investigate the synteny relationships of the CCT genes, we used *G. max*, which has 116 CCT superfamily genes, as a model to construct comparative allograms of *G. max* at the genome level. Here, we selected three model plants for analysis: *O. sativa*, *A. thaliana*, and *V. vinifera*. The Multiple Collinearity Scan toolkit was used to identify orthologous genes between the genomes of the selected plant species (Figure 5). A total of 11, 28 and 27 orthologous gene pairs between the Glyma CCTs and other CCT genes in *O. sativa*, *A. thaliana*, and *V. vinifera* were identified, respectively. Some CCT genes had at least three pairs of orthologous genes, such as Glyma.10G048100.8.p, which may play a crucial role in the evolution of CCT genes. These results show that the CCT genes in *G. max* may originate from the orthologous genes of other plant species.

**Figure 6:**
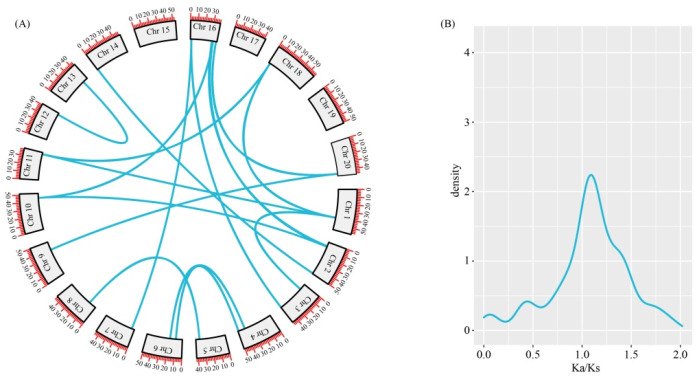
Intraspecific collinearity analysis. (**A**). A collinearity analysis of CCT genes in *G. max*. The blue lines indicate the CCT gene pairs in collinear blocks. (**B**). Ka/Ks ratio of 16 CCT gene pairs.

To investigate whether *G. max* is diploid with a large genome, we further investigated the duplication events in the CCT gene family. The *G. max* genome contains 16 CCT gene pairs that are involved in duplication events (Figure 6A), which are located on different chromosomes, indicating that the expansion of the CCT gene family in *G. max* likely depended mainly on WGD or segmental duplication within genomes. The Ka/Ks allowed estimation of the duplicated CCT genes’ evolutionary selection pressure in *G. max*. We calculated the non-synonymous (Ka), synonymous substitution (Ks), and Ka/Ks ratio of the 16 CCT gene pairs (Figure 6B). A value of Ka/Ks = 1 means that a gene is under neutral selection; Ka/Ks < 1 indicates negative purifying selection, and Ka/Ks > 1 is evidence of positive selection [32]. From 16 pairs of *G. max* CCT genes with a collinear relationship, the Ka/Ks values of 4 gene pairs were calculated to be less than 1, indicating that the CCT genes have undergone a strong negative purifying selection pressure. These results demonstrate the conserved evolution of the CCT genes.

### 3.4. Expression Patterns of CCTs in Plants

**Figure 7:**
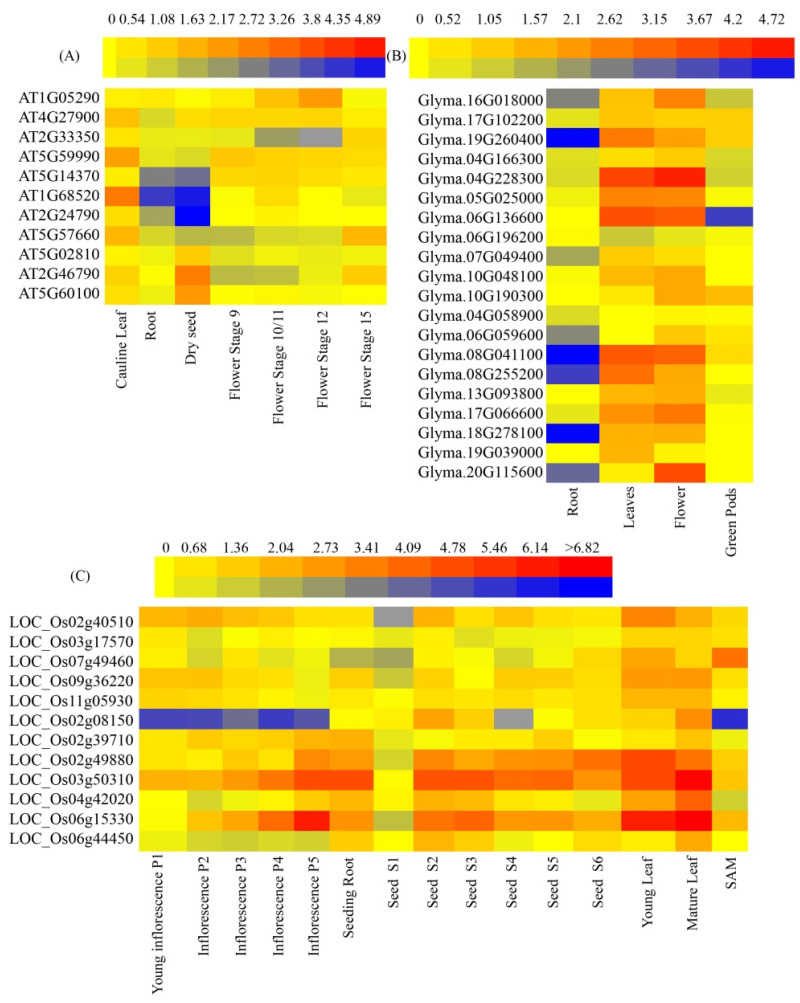
Heat map of the CCT gene expression profiles in various tissues and at different developmental stages of the representative species: (**A**–**C**) expression of CCT genes in *A. thaliana*, *G. max* (soybean), and *O. sativa* (rice), respectively. The red color is upregulated expression, and blue color is downregulated expression.

To investigate the function of CCTs in growth and development, the gene expression profiles of the CCTs were analyzed across different tissue types (e.g., flower, leaf, root, green pod, seed and SAM), from *A. thaliana*, *G. max*, and *O. sativa*, to generate high-resolution spatial expression profiles throughout the plant life cycle (Figure 7).

Significant differences were observed in the expression profiles of the CCT genes in different tissues and at different developmental stages of the three species (Figure 7). For the CCT genes in *A. thaliana*, the highest expression level was detected in the cauline leaf, while they were weakly expressed in the seed, indicating that the CCT genes have spatial expression patterns (Figure 7A). The spatial expression pattern was also found in *G. max*; as we can see, the weakly expressed genes were found in the root, such as Glyma.19G260400, Glyma.08G041100, and Glyma.18G278100, while the highest expression level was detected in the flower (Figure 7B). There is a commonality in the expression of the CCT genes in inflorescence at different developmental stages in *O. sativa*. We found that LOC_Os02g08150 was significantly downregulated throughout the entire stage of inflorescence development. Interestingly, the expression levels of LOC_Os03g50310 and LOC_Os06g15330 increased as the inflorescence matured, and these two genes were also found to have higher expression levels in seeds S2–S6, indicating that they may play a role in inflorescence and seed development. The expression pattern of CCT genes was similar in the young leaf and old leaf of *O. sativa*, with both LOC_Os06g15330 and LOC_Os03g50310 showing high expression. However, LOC_Os02g49880 was observed to be significantly upregulated in only the young leaf, indicating that the it may play a role in the early stage of leaf development. A similar expression pattern was found in the *G. max* leaf, with significantly upregulated expression of Glyma.04G228300, Glyma.06G136600, and Glyma.08G041100. However, no significantly upregulated CCT genes were found in the *A. thaliana* leaf, indicating species differences in the CCT gene expression. When it comes to the flower, similarities between the expression patterns of CCT in the *G. max* leaves and flowers were found, with Glyma.04G228300, Glyma.06G136600, and Glyma.08G041100 being significantly upregulated. However, the expression of Glyma.20G115600 showed significant differences, only being upregulated in the flower, demonstrating the tissue-specific expression of the CCT gene. All in all, these results suggested that the CCT genes displayed diverse tissue-specific expression patterns in different species, and they provided a theoretical foundation for further exploration of the biological functions of CCT genes.

### 3.5. Analysis of CCT Protein–Protein Interaction

**Figure 8:**
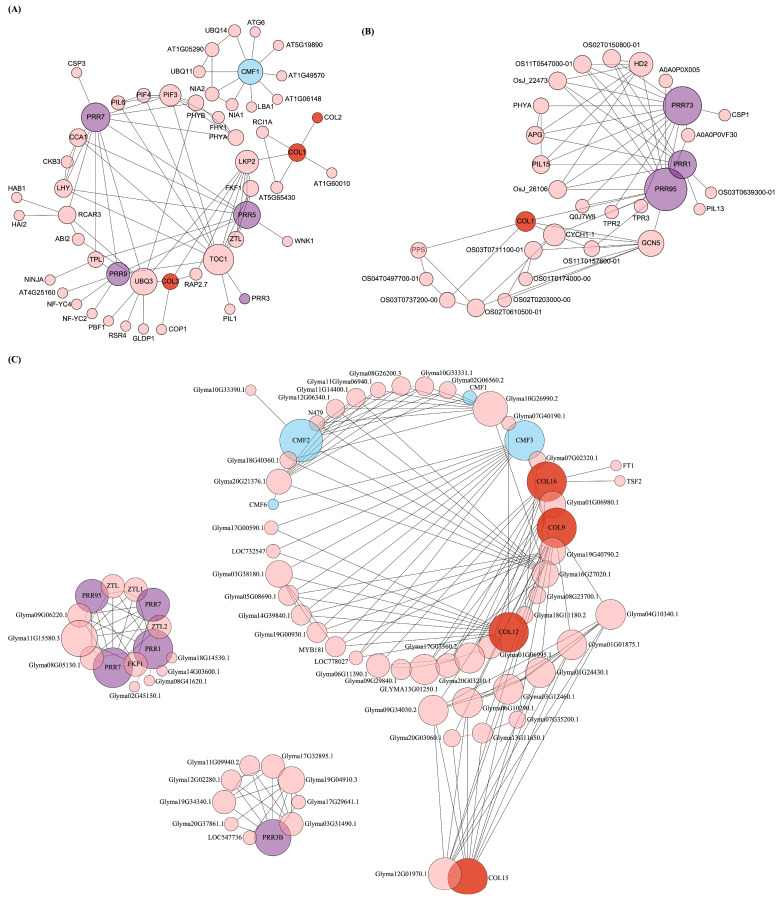
Global representation of the predicted protein–protein interaction network of the CCT proteins. (**A**–**C**) Protein–protein interaction network of the CCT proteins from *A. thaliana*, *O. sativa*, and *G. max*, respectively. The proteins are represented by nodes (circles), and the interactions are represented by edges (lines) connecting nodes. The number of degrees is represented by the node size.

The study of interaction networks can help us better understand the biological processes and molecular pathways involving proteins. Analysis of CCT protein–protein interactions provides important details about the as yet unidentified function of CCT. Based on the orthoVenn2 analysis and evolutionary analysis, we constructed protein interaction network relationship diagrams of the CCT proteins from *A. thaliana*, *O. sativa*, and *G. max* (Figure 8A–C). As shown, the CCT proteins interact in different ways with the regulatory proteins. We observed that the PRR family proteins tend to interact with the PHY family proteins, which were known to be involved in lifelong plant morphogenesis, including seed germination, flowering, fruiting, and senescence. The interaction between the COL family proteins from *G. max* and the MYB family transcription factors may help to regulate the growth, development, and physiological metabolism in plants.

## 4. Discussion

It is known that members of the CCT superfamily play crucial roles in flowering, development, circadian rhythms, and abiotic stress tolerance [33,34]. According to the functional domains contained in genes, the CCT superfamily is divided into the PRR (PSEUDO-RESPONSE REGULATOR) sub-family, COL (CONSTANS-LIKE) sub-family, CMF (CCT MOTIF FAMILY) sub-family or CCT domain-containing proteins, which are prevalent in a wide range of flowering plants and play a key role as specific transcriptional regulators in integrating environmental signals and regulating the reproduction progress [35]. CCT genes have been identified and characterized in terrestrial plants, but the CCT superfamily in photosynthetic organisms has not yet been systematically and comprehensively studied. Here, a total of 700 CCT genes were identified across 30 species, as detailed in Table 1. These genes were subsequently categorized into three sub-families based on the presence of their conserved motifs. A total of 408 CCT genes belong to the PRR sub-family, 189 CCT genes belong to the COL sub-family, and 103 CCT genes belong to the CMF sub-family. Furthermore, consistent with the classification results of the CCT genes based on different domains and specific CCT domain splitting, the 700 CCT genes were categorized into three discrete groups in the phylogenetic analyses (Figure 1, Figure 2 and Figure 3). The CCT domain in the CCT family of different plants is highly conserved [36]. There are a few exceptions in the CCT proteins of *G. max*, specifically in Glyma.10G048100.1.p, Glyma.13G135900.1.p, and Glyma.13G135900.2.p from the PRR sub-family, where an Arg-Glu-Arg sequence is inserted between the 15th and 16th amino acid positions in their CCT domains, diverging from the other CCT domains. As revealed by the topological structure of the evolutionary trees, the CCT genes from all the terrestrial plants are typically conserved and share similar patterns in terms of protein domains and motifs. Based on the variances in both the quantity and organization of conserved motifs, which serve as the distinguishing features among the groups, we believe that the genetic distance between algal CCTs and terrestrial plant CCTs is far apart. Among the algal plants, brown algae, recognized for their ability to synthesize fucoidan, possess a distinctive circadian rhythm mechanism (brown clock) like diatoms. Despite the fact that brown algae and diatoms exhibit a reduced number of PRR genes compared to higher plants, they express proteins with structural domains similar to animal clock components, including basic Helix-Loop-Helix PER-ARNT-SIM (bHLH-PAS) proteins and specialized photoreceptors [37].

Gene duplication leads to the divergence of gene families, which is another key event that occurs during the evolutionary process [38,39,40]. Gene replication events related to the CMF gene were detected via genome-wide replication analysis and phylogenetic analysis. Synteny analysis of the CCT superfamily genes in *G. max*, *O. sativa*, *A. thaliana*, and *V. vinifera* revealed that some genes, such as Glyma.10G048100.8.p, had three gene copies tandemly arranged in each sub-genome of *G. max* and *V. vinifera* (Figure 5). Furthermore, variations in the quantity of the CCT members across the superfamily and sub-families of plants suggested that they may have undergone different evolution trajectories. An examination of the selective pressures on *G. max* indicated that the CCT gene family had diverged in their evolutionary paths, a consequence of varied selective forces throughout the evolution process, leading to a heterogeneous population. Our findings indicate that the mean Ka/Ks ratio for the 16 CCT gene pairs in *G. max* was greater than 1, implying that the gene family predominantly experienced positive selection. However, four genes had Ka/Ks ratios less than 1, which suggests a partial impact of negative selection on the gene family [32,41]. Post-gene fragment replication, strong purifying selection followed. These findings indicate that the structure of the CCT genes is relatively conserved.

The gene expression profiling revealed a tissue-specific pattern for the CCT genes, with a notable variation in the expression levels predominantly observed among genes from different sub-families. An increase in the gene family’s size within a species correlates with a pronounced specialization in their expression profiles. In the case of *G. max*, the CCT family gene Glyma.20G115600 had the highest expression in flowers and the lowest expression in roots. This differential expression aligned with the fact that CCT is a photoperiodic flowering regulatory gene [1,2]. An interesting phenomenon has been observed, where the expression of AT1G68520 in the COL sub-family is downregulated in the root and seed of *A. thaliana*. Similarly, in the root of *G. max*, decreased expression of genes such as Glyma.08G041100, Glyma.08G255200, and Glyma-18G278100 was observed, which also belong to the COL sub-family. On the contrary, the expression levels of the genes LOC_Os03g50310 and LOC_Os06g15330 were upregulated in the inflorescence, seed, and leave of *O. sativa*, which also belong to the COL sub-family. The differences in this expression pattern revealed the diverse roles of the COL sub-family genes in different plant organs and growth stages, which may involve the adaptive response of plants to environmental changes and key regulatory processes of growth and development. Several studies have demonstrated that CCTs can also regulate diverse developmental processes through interactions with other proteins, such as the interaction between the CCA1 clock component and PRR1, which can regulate circadian-associated biological events in *Arabidopsis*. In *Arabidopsis*, the physical interactions among PRR9, PRR7, and PRR5 with BBX19 and BBX18 follow a precise temporal sequence from dawn to dusk, mirroring the temporal pattern of PRRs’ accumulation [42,43].

## 5. Conclusions

In short, a total of 700 CCTs were identified in 30 plant species and systematically grouped into three sub-families based on the phylogenetic relationships, conserved domains, and conserved motifs. The evolutionary trajectory of the CCT genes is marked by large-scale indirect amplification and gene deletion, as evidenced by the analysis of the whole-genome replication events. *G. max* stands out with its large CCT gene pool, exemplifying this evolutionary pattern. Selective pressures within the CCT genes were evident, indicating a conserved evolutionary mechanism in the CCT gene family. The expression profiles indicated diverse CCT functions, with tissue-specific differential expression of the CCT genes. These findings provide a comprehensive overview of the CCT family in plants, providing a robust foundation for future investigations into CCT gene functions, thereby enriching our understanding of plant biology and potential applications in agriculture.

## Figures and Tables

**Table 1:** Distribution of the members of the CCT gene family in 30 plant species.

Taxa	Species	Total	PRR	COL	CMF
Eudicot	*Arabidopsis thaliana*	13	5	3	5
	*Brassica rapa*	30	9	15	6
	*Citrus sinensis*	30	23	6	1
	*Gossypium darwinii*	48	22	16	10
	*Gossypium hirsutum*	117	58	26	33
	*Glycine max*	116	63	18	35
	*Medicago truncatula*	31	22	6	3
	*Populus trichocarpa*	38	23	10	5
	*Solanum lycopersicum*	17	6	9	2
	*Theobroma cacao*	27	18	7	2
	*Vitis vinifera*	28	21	6	1
Bacillariophyta	*Cylindrotheca closterium*	1	1	0	0
	*Mayamaea pseudoterrestris*	1	1	0	0
	*Nitzschia inconspicua*	4	4	0	0
	*Phaeodactylum tricornutum CCAP 1055/1*	2	2	0	0
	*Seminavis robusta*	2	2	0	0
Chlorophyta	*Chlamydomonas reinhardtii*	2	2	0	0
	*Volvox cartei*	3	3	0	0
Phaeophyta	*Ectocarpus siliculosus*	1	1	0	0
	*Ectocarpus* sp. *CCAP 1310/34*	2	2	0	0
Monocot	*Hordeum vulgare*	20	7	13	0
	*Oryza sativa*	21	14	7	0
	*Sorghum bicolor*	18	9	9	0
	*Triticum aestivum*	38	33	5	0
	*Zea mays*	17	7	10	0
Moss	*Ceratodon purpureus*	7	5	2	0
	*Sphagnum magellanicum*	17	13	4	0
Fern	*Ceratopteris richardii*	34	23	11	0
Liverwort	*Marchantia polymorpha*	9	6	3	0
Basal angiosperm	*Amborella trichopoda*	6	3	3	0

## Data Availability

Data are contained within the article and Supplementary Materials.

## Supplementary Materials

The following supporting information can be downloaded at https://www.mdpi.com/article/10.3390/genes15070941/s1, Figure S1: Domains of the CCT proteins; Figure S2: Motifs of the CCT proteins; Figure S3: Motifs of the proteins; Table S1: Location information of the 700 CCT genes; Table S2: Conserved motif sequence of all the obtained CCT genes from 30 species.
