# Supplementary material for: Genome-Wide Identification and Characterization of CCT Gene Family from Microalgae to Legumes
Source: Genes (Basel). 2024 Jul 18;15(7):941. doi: 10.3390/genes15070941 (PMC11275407; doi:10.3390/genes15070941)
Supplement: Supplementary file 1 [file genes-15-00941-s001.zip › genes-3072377-supplementary.pdf]

Table S1 Location information of 700 CCT genes

Gene_ID	Chr	Location (start..end)	Species	Class
AmTr_v1.0_scaffold00009.431	scaffold00009	9236313..9243447	*Amborella trichopoda*	PRR
AmTr_v1.0_scaffold00006.243	scaffold00006	8039092..8041532	*Amborella trichopoda*	COL
AmTr_v1.0_scaffold00019.214	scaffold00019	3408669..3410390	*Amborella trichopoda*	COL
AmTr_v1.0_scaffold00073.16	scaffold00073	620001-621272	*Amborella trichopoda*	COL
AmTr_v1.0_scaffold00023.165	scaffold00023	1179543..1179677	*Amborella trichopoda*	PRR
AT1G68520.1	1	25708175..25710989	*Arabidopsis thaliana*	COL
AT2G24790	2	10566959..10567946	*Arabidopsis thaliana*	COL
AT5G57660.1	5	23355573..23356729	*Arabidopsis thaliana*	COL
AT2G33350.5	2	14134116..14136836	*Arabidopsis thaliana*	CMF
AT1G05290.1	1	1539152..1540546	*Arabidopsis thaliana*	CMF
AT4G27900	4	13890858..13892777	*Arabidopsis thaliana*	CMF
AT5G59990.1	5	24151206..24153084	*Arabidopsis thaliana*	CMF
AT5G14370.1	5	4632147..4633651	*Arabidopsis thaliana*	CMF
AtPRR7_At5g02810	5	637681..642026	*Arabidopsis thaliana*	PRR
AtPRR5_At5g24470	5	8356204..8358546	*Arabidopsis thaliana*	PRR
AtPRR1_At5g61380	5	24674963..24678550	*Arabidopsis thaliana*	PRR
AtPRR3_At5g60100	5	24197998..24201364	*Arabidopsis thaliana*	PRR
AtPRR9_At2g46790	2	19232607..19235179	*Arabidopsis thaliana*	PRR
Brara.B00066.1.p	A02	365269..368747	*Brassica rapa*	PRR
Brara.A02750.1.p	A02	23261467..23262883	*Brassica rapa*	CMF
Brara.D02027.1.p	A04	16904297..16907620	*Brassica rapa*	CMF
Brara.E01059.1.p	A05	6212564..6214268	*Brassica rapa*	CMF
Brara.H03045.1.p	A08	22820544..22823639	*Brassica rapa*	CMF
Brara.I05454.1.p	A09	43935688..43937097	*Brassica rapa*	CMF
Brara.J00262.1.p	A10	1460914..1462483	*Brassica rapa*	CMF
Brara.C04252.1.p	A03	22379943..22383006	*Brassica rapa*	PRR
Brara.E00247.1.p	A05	1387295..1389185	*Brassica rapa*	PRR
Brara.F02666.1.p	A06	22010751..22013645	*Brassica rapa*	PRR
Brara.I00569.1.p	A09	3176223..3179357	*Brassica rapa*	PRR
Brara.I00658.1.p	A09	3664785..3666402	*Brassica rapa*	PRR
Brara.J01369.1.p	A10	12254627..12257720	*Brassica rapa*	PRR
Brara.J01369.2.p	A10	12254627..12257720	*Brassica rapa*	PRR
Brara.J02854.1.p	A10	19511223..19515201	*Brassica rapa*	PRR
Brara.A03895.1.p	A01	30678761..30679726	*Brassica rapa*	COL
Brara.B00625.1.p	A02	2917228..2918175	*Brassica rapa*	COL
Brara.B01196.1.p	A02	6043129..6044670	*Brassica rapa*	COL
Brara.B01797.1.p	A02	10617685..10619372	*Brassica rapa*	COL
Brara.D01502.1.p	A04	13503708..13504860	*Brassica rapa*	COL
Brara.E01711.1.p	A05	12283954..12285455	*Brassica rapa*	COL
Brara.F02700.1.p	A06	22315737..22317488	*Brassica rapa*	COL
Brara.G00872.1.p	A07	10525367..10527053	*Brassica rapa*	COL
Brara.G02520.1.p	A07	20957285..20959108	*Brassica rapa*	COL

Brara.H02107.1.p	A08	18807916..18809699	*Brassica rapa*	COL
Brara.I00559.1.p	A09	3108494..3110325	*Brassica rapa*	COL
Brara.I03083.1.p	A09	29763002..29764988	*Brassica rapa*	COL
Brara.J01176.1.p	A10	11152494..11154003	*Brassica rapa*	COL
Brara.J01926.1.p	A10	15374341..15375919	*Brassica rapa*	COL
Brara.J01927.1.p	A10	15384101..15385684	*Brassica rapa*	COL
CepurR40.4G227400.1.p	4	19028955..19032858	*Ceratodon purpureus*	COL
CepurR40.9G121400.1.p	9	10728032..10730557	*Ceratodon purpureus*	COL
CepurR40.5G102900.1.p	12	9869769..9875805	*Ceratodon purpureus*	PRR
CepurR40.5G102900.2.p	6	9869769..9875805	*Ceratodon purpureus*	PRR
CepurR40.5G102900.3.p	6	9869769..9875805	*Ceratodon purpureus*	PRR
CepurR40.5G102900.4.p	6	9869769..9875805	*Ceratodon purpureus*	PRR
CepurR40.5G103300.1.p	16	9881843..9889341	*Ceratodon purpureus*	PRR
Ceric.04G018800.1.p	4G	29985422..29992066	*Ceratopteris richardii*	PRR
Ceric.04G018800.2.p	4G	29985419..29992120	*Ceratopteris richardii*	PRR
Ceric.04G018800.3.p	4G	29985416..29992068	*Ceratopteris richardii*	PRR
Ceric.04G018800.4.p	4G	29985516…29992066	*Ceratopteris richardii*	PRR
Ceric.04G018800.5.p	4G	29985446..29991968	*Ceratopteris richardii*	PRR
Ceric.04G018800.6.p	4G	29985449..29991941	*Ceratopteris richardii*	PRR
Ceric.04G018800.7.p	4G	29985525..29991929	*Ceratopteris richardii*	PRR
Ceric.04G094100.1.p	4G	177765561..177851061	*Ceratopteris richardii*	PRR
Ceric.04G094100.2.p	4G	177765506..177850917	*Ceratopteris richardii*	PRR
Ceric.05G086900.1.p	5G	168894499..168905273	*Ceratopteris richardii*	PRR
Ceric.05G086900.2.p	5G	168894614..168905118	*Ceratopteris richardii*	PRR
Ceric.05G086900.3.p	5G	168894605..168904685	*Ceratopteris richardii*	PRR
Ceric.05G086900.4.p	5G	168894605..168904201	*Ceratopteris richardii*	PRR
Ceric.04G058700.1.p	04G	103895264..103896416	*Ceratopteris richardii*	COL
Ceric.12G022400.1.p	12G	46889790..46894542	*Ceratopteris richardii*	COL
Ceric.12G022400.2.p	12G	46890067..46894542	*Ceratopteris richardii*	COL
Ceric.12G022400.3.p	12G	46890031..46894195	*Ceratopteris richardii*	COL
Ceric.12G022400.4.p	12G	46890067..46893607	*Ceratopteris richardii*	COL
Ceric.20G041500.1.p	20G	74898362..74899611	*Ceratopteris richardii*	COL
Ceric.27G026400.1.p	27G	75209664..75215239	*Ceratopteris richardii*	COL
Ceric.27G026400.2.p	27G	75209664..75215239	*Ceratopteris richardii*	COL
Ceric.27G026400.3.p	27G	75209696..75214197	*Ceratopteris richardii*	COL
Ceric.27G026400.4.p	27G	75209717..75214141	*Ceratopteris richardii*	COL
Ceric.27G026400.6.p	27G	75209717..75213899	*Ceratopteris richardii*	COL
Ceric.08G065800.1.p	8G	175184089..175190298	*Ceratopteris richardii*	PRR
Ceric.08G065800.3.p	8G	175184171..175190152	*Ceratopteris richardii*	PRR
Ceric.08G065800.4.p	8G	175184171…175190152	*Ceratopteris richardii*	PRR
Ceric.08G065800.5.p	8G	175184233..175190152	*Ceratopteris richardii*	PRR
Ceric.08G065800.6.p	8G	175184178..175190019	*Ceratopteris richardii*	PRR
Ceric.24G072900.1.p	24G	139660864..139765246	*Ceratopteris richardii*	PRR
Ceric.24G072900.2.p	24G	139660907..139765084	*Ceratopteris richardii*	PRR

Ceric.26G022500.1.p	26G	63599199..63603903	*Ceratopteris richardii*	PRR
Ceric.26G022500.2.p	26G	63599859..63603567	*Ceratopteris richardii*	PRR
Ceric.26G022500.3.p	26G	63599326..63603842	*Ceratopteris richardii*	PRR
Cre02.g094150.t1.2	chromosome_2	2779156..2784937	*Chlamydomonas reinhardtii*	PRR
Cre16.g676421.t1.1	chromosome_16	6879565..6890117	*Chlamydomonas reinhardtii*	PRR
orange1.1g003994m	scaffold00172	153239…161720	*Citrus sinensis*	PRR
orange1.1g003995m	scaffold00172	153239..161720	*Citrus sinensis*	PRR
orange1.1g004001m	scaffold00172	153239..161720	*Citrus sinensis*	PRR
orange1.1g004184m	scaffold00172	153239..161720	*Citrus sinensis*	PRR
orange1.1g005631m	scaffold00172	153239..161720	*Citrus sinensis*	PRR
orange1.1g005920m	scaffold00343	171416..176919	*Citrus sinensis*	PRR
orange1.1g007196m	scaffold00172	153239..161720	*Citrus sinensis*	PRR
orange1.1g007205m	scaffold00172	153239..161720	*Citrus sinensis*	PRR
orange1.1g007462m	scaffold00343	171416..176919	*Citrus sinensis*	PRR
orange1.1g008649m	scaffold00111	52047..57632	*Citrus sinensis*	PRR
orange1.1g008655m	scaffold00111	52047..57632	*Citrus sinensis*	PRR
orange1.1g008761m	scaffold00111	52047..57632	*Citrus sinensis*	PRR
orange1.1g010665m	scaffold00343	171416..176919	*Citrus sinensis*	PRR
orange1.1g011192m	scaffold00343	171416..176919	*Citrus sinensis*	PRR
orange1.1g011208m	scaffold00343	171416..176919	*Citrus sinensis*	PRR
orange1.1g012558m	scaffold00008	688769..690765	*Citrus sinensis*	COL
orange1.1g015643m	scaffold00090	61980..63788	*Citrus sinensis*	COL
orange1.1g017166m	scaffold00048	680027..681613	*Citrus sinensis*	COL
orange1.1g017738m	scaffold00030	427507..429256	*Citrus sinensis*	COL
orange1.1g017819m	scaffold00071	12696..14709	*Citrus sinensis*	COL
orange1.1g019028m	scaffold00025	698938..700909	*Citrus sinensis*	COL
orange1.1g011217m	scaffold00343	17141.. 176919	*Citrus sinensis*	PRR
orange1.1g042408m	scaffold00512	84853..87139	*Citrus sinensis*	CMF
orange1.1g013244m	scaffold00343	17141.. 176919	*Citrus sinensis*	PRR
orange1.1g013329m	scaffold00111	52047..57632	*Citrus sinensis*	PRR
orange1.1g017013m	scaffold00111	52047..57632	*Citrus sinensis*	PRR
orange1.1g018624m	scaffold00343	171416…176919	*Citrus sinensis*	PRR
orange1.1g038567m	scaffold00014	981622..983634	*Citrus sinensis*	PRR
orange1.1g039762m	scaffold00024	1261874..1263806	*Citrus sinensis*	PRR
orange1.1g042954m	scaffold00001	3390805..3395427	*Citrus sinensis*	PRR
CAJ1930941.1	Ccl_contig11	2253..2558	*Cylindrotheca closterium*	PRR
CBN75530.1	scaffold sctg_114	54293..61367	*Ectocarpus siliculosus*	PRR
CAB1113560.1	ESUB_scaffold464	8188..11450	*Ectocarpus* sp. CCAP 1310/34	PRR
CAB1107368.1	ESUB_scaffold264	9837..19275	*Ectocarpus* sp. CCAP 1310/34	PRR
Glyma.04G058900.1.p	4	4821349..4823038	*Glycine max*	COL

Glyma.10G134400.1.p	10	36188879..36193749	*Glycine max*	CMF
Glyma.10G134400.2.p	10	36191064..36193815	*Glycine max*	CMF
Glyma.10G190300.1.p	10	42408882..42413232	*Glycine max*	CMF
Glyma.10G190300.2.p	10	42408900..42411596	*Glycine max*	CMF
Glyma.12G196100.10.p	12	37190017..37194670	*Glycine max*	CMF
Glyma.12G196100.11.p	12	37190017..37194670	*Glycine max*	CMF
Glyma.12G196100.12.p	12	37190017..37194670	*Glycine max*	CMF
Glyma.12G196100.13.p	12	37190017..37194670	*Glycine max*	CMF
Glyma.12G196100.14.p	12	37190355..37194651	*Glycine max*	CMF
Glyma.12G196100.2.p	12	37190307..37194649	*Glycine max*	CMF
Glyma.12G196100.8.p	12	37190017..37194670	*Glycine max*	CMF
Glyma.12G196100.9.p	12	37190017..37194672	*Glycine max*	CMF
Glyma.13G306400.10.p	13	37190017..37194670	*Glycine max*	CMF
Glyma.13G306400.11.p	13	39629255..39633560	*Glycine max*	CMF
Glyma.13G306400.12.p	13	39629255..39633917	*Glycine max*	CMF
Glyma.13G306400.13.p	13	39629255..39633918	*Glycine max*	CMF
Glyma.13G306400.14.p	13	39629255..39633917	*Glycine max*	CMF
Glyma.13G306400.15.p	13	39629255..39633922	*Glycine max*	CMF
Glyma.13G306400.16.p	13	39629255..39633560	*Glycine max*	CMF
Glyma.13G306400.17.p	13	39629255..39633917	*Glycine max*	CMF
Glyma.13G306400.18.p	13	39629255..39633981	*Glycine max*	CMF
Glyma.13G306400.19.p	13	39629255..39633981	*Glycine max*	CMF
Glyma.13G306400.20.p	13	39629255..39633917	*Glycine max*	CMF
Glyma.13G306400.21.p	13	39629255..39633560	*Glycine max*	CMF
Glyma.13G306400.23.p	13	39629255..39633981	*Glycine max*	CMF
Glyma.05G233700.1.p	5	41129378..41131198	*Glycine max*	COL
Glyma.06G059600.1.p	6	4497348..4498800	*Glycine max*	COL
Glyma.06G059600.2.p	6	4497348..4498632	*Glycine max*	COL
Glyma.07G091400.1.p	7	8392215..8394595	*Glycine max*	COL
Glyma.08G041100.1.p	8	3252455..3254458	*Glycine max*	COL
Glyma.08G255200.1.p	8	22663358..22665994	*Glycine max*	COL
Glyma.09G184600.1.p	9	41877396..41879933	*Glycine max*	COL
Glyma.09G184600.2.p	9	41877396..41879933	*Glycine max*	COL
Glyma.09G184600.3.p	9	41877396..41879933	*Glycine max*	COL
Glyma.10G274300.1.p	10	49788414..49791139	*Glycine max*	COL
Glyma.13G050300.1.p	13	13695600..13698195	*Glycine max*	COL
Glyma.13G093800.1.p	13	19927838..19929743	*Glycine max*	COL
Glyma.17G066600.1.p	17	5148779..5150706	*Glycine max*	COL
Glyma.18G278100.1.p	18	56236008..56238724	*Glycine max*	COL
Glyma.18G278100.2.p	18	56236008..56238724	*Glycine max*	COL
Glyma.19G039000.1.p	19	5457432..5460114	*Glycine max*	COL
Glyma.20G115600.1.p	20	35710259..35712406	*Glycine max*	COL
Glyma.13G135900.2.p	13	23930419..23941569	*Glycine max*	PRR
Glyma.13G135900.5.p	13	23930440..23941569	*Glycine max*	PRR

Glyma.13G135900.6.p	13	23930255..23941601	*Glycine max*	PRR
Glyma.13G135900.7.p	13	23930419..23941557	*Glycine max*	PRR
Glyma.13G135900.8.p	13	23930297..23941557	*Glycine max*	PRR
Glyma.13G135900.9.p	13	23930407..23941569	*Glycine max*	PRR
Glyma.16G018000.1.p	16	1590112..1595644	*Glycine max*	PRR
Glyma.16G018000.2.p	16	1590112..1594729	*Glycine max*	PRR
Glyma.17G102200.1.p	17	8016979..8022153	*Glycine max*	PRR
Glyma.17G102200.5.p	17	8017278..8022163	*Glycine max*	PRR
Glyma.19G260400.1.p	19	50890562..50895522	*Glycine max*	PRR
Glyma.19G260400.2.p	19	50890571..50895525	*Glycine max*	PRR
Glyma.19G260400.3.p	19	50890571..50895525	*Glycine max*	PRR
Glyma.19G260400.4.p	19	50890563..50895522	*Glycine max*	PRR
Glyma.19G260400.6.p	19	50890686..50895516	*Glycine max*	PRR
Glyma.19G260400.7.p	19	50890556..50895523	*Glycine max*	PRR
Glyma.19G260400.8.p	19	50890680..50895501	*Glycine max*	PRR
Glyma.03G261300.1.p	3	46654181..46659270	*Glycine max*	PRR
Glyma.03G261300.2.p	3	46654206..46658981	*Glycine max*	PRR
Glyma.03G261300.4.p	3	46654329…46659270	*Glycine max*	PRR
Glyma.03G261300.5.p	3	46654329..46658273	*Glycine max*	PRR
Glyma.04G166300.1.p	4	40573999..40579558	*Glycine max*	PRR
Glyma.04G166300.2.p	4	40573998..40579763	*Glycine max*	PRR
Glyma.04G166300.3.p	4	40573991..40579497	*Glycine max*	PRR
Glyma.04G166300.4.p	4	40574024..40579530	*Glycine max*	PRR
Glyma.04G228300.1.p	4	48579364..48584883	*Glycine max*	PRR
Glyma.04G228300.10.p	4	48579432..48584205	*Glycine max*	PRR
Glyma.04G228300.3.p	4	48579438..48584460	*Glycine max*	PRR
Glyma.04G228300.6.p	4	48579302..48584205	*Glycine max*	PRR
Glyma.04G228300.7.p	4	48579371..48584293	*Glycine max*	PRR
Glyma.04G228300.8.p	4	48578926..48584090	*Glycine max*	PRR
Glyma.04G228300.9.p	4	48579300..48584466	*Glycine max*	PRR
Glyma.05G025000.1.p	5	2179652..2184473	*Glycine max*	PRR
Glyma.05G025000.3.p	5	2179652..2184519	*Glycine max*	PRR
Glyma.06G136600.1.p	6	11138091..11143177	*Glycine max*	PRR
Glyma.06G136600.3.p	6	11138050..11142743	*Glycine max*	PRR
Glyma.06G196200.1.p	6	17550832..17556959	*Glycine max*	PRR
Glyma.07G049400.16.p	7	4185541..4190036	*Glycine max*	PRR
Glyma.07G049400.18.p	7	4184806..4189950	*Glycine max*	PRR
Glyma.07G049400.19.p	7	4185488..4190147	*Glycine max*	PRR
Glyma.07G049400.2.p	7	4185490..4190097	*Glycine max*	PRR
Glyma.07G049400.20.p	7	4184806..4190066	*Glycine max*	PRR
Glyma.07G049400.21.p	7	4185489..4190094	*Glycine max*	PRR
Glyma.07G049400.22.p	8	4185298..4190089	*Glycine max*	PRR
Glyma.07G049400.23.p	7	4185484..4190089	*Glycine max*	PRR
Glyma.07G049400.4.p	7	4185489..4190147	*Glycine max*	PRR

Glyma.07G049400.8.p	7	4185480..4190120	*Glycine max*	PRR
Glyma.07G049400.9.p	7	4185481..4190089	*Glycine max*	PRR
Glyma.10G048100.1.p	10	4282985..4294879	*Glycine max*	PRR
Glyma.10G048100.10.p	10	4283080..4294879	*Glycine max*	PRR
Glyma.10G048100.11.p	10	4283100..4294879	*Glycine max*	PRR
Glyma.10G048100.12.p	10	4283082..4294925	*Glycine max*	PRR
Glyma.10G048100.13.p	10	4283080..4294879	*Glycine max*	PRR
Glyma.10G048100.3.p	10	4282855..4294542	*Glycine max*	PRR
Glyma.10G048100.4.p	10	4283080..4294879	*Glycine max*	PRR
Glyma.10G048100.5.p	10	4283113..4294879	*Glycine max*	PRR
Glyma.10G048100.6.p	10	4283114..4294948	*Glycine max*	PRR
Glyma.10G048100.7.p	10	4282985..4294879	*Glycine max*	PRR
Glyma.10G048100.8.p	10	4283082..4294879	*Glycine max*	PRR
Glyma.10G048100.9.p	10	4283079..4294879	*Glycine max*	PRR
Glyma.13G135900.1.p	13	23930395..23941562	*Glycine max*	PRR
Glyma.13G135900.10.p	13	23930452..23941557	*Glycine max*	PRR
Glyma.13G135900.11.p	13	23930510..23941570	*Glycine max*	PRR
Glyma.13G306400.24.p	Gm13	39630853..39633412	*Glycine max*	CMF
Glyma.13G306400.25.p	Gm13	39630853..39633412	*Glycine max*	CMF
Glyma.13G306400.26.p	Gm13	39630853..39633412	*Glycine max*	CMF
Glyma.13G306400.27.p	Gm13	39630853..39633412	*Glycine max*	CMF
Glyma.13G306400.5.p	Chr13	40258529..40261088	*Glycine max*	CMF
Glyma.13G306400.6.p	Chr13	40258529..40261088	*Glycine max*	CMF
Glyma.20G085100.1.p	Chr20	31775084..31779069	*Glycine max*	CMF
Glyma.20G085100.2.p	Chr20	31776902..31779069	*Glycine max*	CMF
Glyma.20G200400.1.p	Chr20	43782678..43784869	*Glycine max*	CMF
Glyma.20G200400.2.p	Chr20	43781391..43784869	*Glycine max*	CMF
Gorai.003G098300.1	3	30484231..30489557	*Gossypium darwinii*	PRR
Gorai.001G039500.1	1	3676945..3678125	*Gossypium darwinii*	COL
Gorai.002G218400.1	2	57033477..57035219	*Gossypium darwinii*	COL
Gorai.003G104600.1	3	32248407..32249974	*Gossypium darwinii*	COL
Gorai.004G030300.1	4	2397135..2398635	*Gossypium darwinii*	COL
Gorai.004G102000.1	4	18921948..18923796	*Gossypium darwinii*	COL
Gorai.004G102000.2	4	18921948..18923796	*Gossypium darwinii*	COL
Gorai.004G113000.1	4	25961240..25963398	*Gossypium darwinii*	COL
Gorai.006G061600.1	6	22449476..22451500	*Gossypium darwinii*	COL
Gorai.006G061600.2	6	22449507..22451500	*Gossypium darwinii*	COL
Gorai.006G061600.3	6	22449507..22451500	*Gossypium darwinii*	COL
Gorai.008G008400.2	8	1001595..1004421	*Gossypium darwinii*	COL
Gorai.005G053500.1	5	5289768..5292114	*Gossypium darwinii*	CMF
Gorai.005G209100.1	5	58989570..58994721	*Gossypium darwinii*	CMF
Gorai.005G209100.2	5	58989570..58994721	*Gossypium darwinii*	CMF
Gorai.006G073000.1	6	29258632..29261148	*Gossypium darwinii*	CMF
Gorai.011G178300.1	11	41985755..41988335	*Gossypium darwinii*	CMF

Gorai.013G071300.1	13	8325222..8330097	*Gossypium darwinii*	CMF
Gorai.013G071300.2	13	8325222..8330097	*Gossypium darwinii*	CMF
Gorai.013G071300.3	13	8325222..8330097	*Gossypium darwinii*	CMF
Gorai.013G071300.4	13	8325222..8330097	*Gossypium darwinii*	CMF
Gorai.013G071300.5	13	8325222..330097	*Gossypium darwinii*	CMF
Gorai.008G059900.1	8	9451492..9453391	*Gossypium darwinii*	COL
Gorai.009G065600.1	9	4656585..4658271	*Gossypium darwinii*	COL
Gorai.010G245200.1	10	61425655..61427048	*Gossypium darwinii*	COL
Gorai.010G245200.2	10	61425559..61427480	*Gossypium darwinii*	COL
Gorai.013G246000.1	13	56481092..56483308	*Gossypium darwinii*	COL
Gorai.003G098300.2	3	30484189..30489639	*Gossypium darwinii*	PRR
Gorai.003G098300.3	3	30484231..30489581	*Gossypium darwinii*	PRR
Gorai.003G098300.4	3	30484950..30489581	*Gossypium darwinii*	PRR
Gorai.006G177800.1	6	43510492..43517222	*Gossypium darwinii*	PRR
Gorai.006G177800.2	6	43510492..43517222	*Gossypium darwinii*	PRR
Gorai.007G017100.1	7	1308179..1312663	*Gossypium darwinii*	PRR
Gorai.007G113000.1	7	8758781..8763097	*Gossypium darwinii*	PRR
Gorai.007G113000.2	7	8758504..8763503	*Gossypium darwinii*	PRR
Gorai.007G113000.3	7	8758781..8763097	*Gossypium darwinii*	PRR
Gorai.007G374800.1	7	60664665..60670240	*Gossypium darwinii*	PRR
Gorai.007G374800.2	7	60664664..60670217	*Gossypium darwinii*	PRR
Gorai.008G265100.1	8	54446660..54449926	*Gossypium darwinii*	PRR
Gorai.008G265100.2	8	54446634..54450113	*Gossypium darwinii*	PRR
Gorai.009G103700.1	9	7501704..7508434	*Gossypium darwinii*	PRR
Gorai.012G001000.1	12	123323..127833	*Gossypium darwinii*	PRR
Gorai.012G092600.1	12	16303688…16310655	*Gossypium darwinii*	PRR
Gorai.012G092600.2	12	16303688..16310756	*Gossypium darwinii*	PRR
Gorai.012G092600.3	12	16303688..16310756	*Gossypium darwinii*	PRR
Gorai.012G092600.4	12	16303688..16310655	*Gossypium darwinii*	PRR
Gorai.012G092600.5	12	16303688..16310655	*Gossypium darwinii*	PRR
Gorai.012G092600.6	12	16303688..16310655	*Gossypium darwinii*	PRR
Gohir.D05G099700.3.p	D05	8245540..8251561	*Gossypium hirsutum*	PRR
Gohir.D09G156300.2.p	D09	45963770..45970541	*Gossypium hirsutum*	PRR
Gohir.D09G156300.6.p	D09	45963770..45970541	*Gossypium hirsutum*	PRR
Gohir.D09G156300.7.p	D09	45963807..45970507	*Gossypium hirsutum*	PRR
Gohir.D11G016300.1.p	D11	1410335..1414180	*Gossypium hirsutum*	PRR
Gohir.D11G016300.3.p	D11	1410335..1414180	*Gossypium hirsutum*	PRR
Gohir.D11G105700.1.p	D11	9492357..9496925	*Gossypium hirsutum*	PRR
Gohir.D11G105700.2.p	D11	9492357..9496925	*Gossypium hirsutum*	PRR
Gohir.A02G042600.1.p	A02	5550610..5553941	*Gossypium hirsutum*	CMF
Gohir.A02G042600.1.p	A02	9009450..9011799	*Gossypium hirsutum*	CMF
Gohir.A03G163400.1.p	A03	104953276..104958432	*Gossypium hirsutum*	CMF
Gohir.A03G163400.3.p	A03	104953276..104958432	*Gossypium hirsutum*	CMF
Gohir.A03G163400.4.p	A03	104953276..104958432	*Gossypium hirsutum*	CMF

Gohir.A09G059500.1.p	A09	54845854..54849516	*Gossypium hirsutum*	CMF
Gohir.A10G109100.1.p	A10	26461707..26466208	*Gossypium hirsutum*	CMF
Gohir.A10G109100.3.p	A10	26461707..26466208	*Gossypium hirsutum*	CMF
Gohir.A13G059300.1.p	A13	12831329..12836040	*Gossypium hirsutum*	CMF
Gohir.A13G059300.2.p	A13	12831329..12836040	*Gossypium hirsutum*	CMF
Gohir.A13G059300.3.p	A13	12831991..12836040	*Gossypium hirsutum*	CMF
Gohir.A13G059300.4.p	A13	12831991..12836040	*Gossypium hirsutum*	CMF
Gohir.A13G059300.5.p	A13	12831329..12836040	*Gossypium hirsutum*	CMF
Gohir.A13G059300.6.p	A13	12831329..12836040	*Gossypium hirsutum*	CMF
Gohir.A13G059300.7.p	A13	12831329..12836040	*Gossypium hirsutum*	CMF
Gohir.D02G048200.1.p	D02	6099975..6102096	*Gossypium hirsutum*	CMF
Gohir.D02G186800.1.p	D02	66704650..66709931	*Gossypium hirsutum*	CMF
Gohir.D02G186800.2.p	D02	66704721..66709931	*Gossypium hirsutum*	CMF
Gohir.D09G058800.1.p	D09	30856668..30860421	*Gossypium hirsutum*	CMF
Gohir.D09G058800.2.p	D09	30856668..30859104	*Gossypium hirsutum*	CMF
Gohir.D10G158700.2.p	D10	46343891..46347268	*Gossypium hirsutum*	CMF
Gohir.D10G158700.3.p	D10	46343638..46347268	*Gossypium hirsutum*	CMF
Gohir.D10G158700.4.p	D10	46342916..46347270	*Gossypium hirsutum*	CMF
Gohir.D13G062900.10.p	D13	8988122..8993084	*Gossypium hirsutum*	CMF
Gohir.D13G062900.11.p	D13	8988122..8993084	*Gossypium hirsutum*	CMF
Gohir.D13G062900.12.p	D13	8988122..8993084	*Gossypium hirsutum*	CMF
Gohir.D13G062900.13.p	D13	8988121..8993085	*Gossypium hirsutum*	CMF
Gohir.D13G062900.3.p	D13	8988122..8993084	*Gossypium hirsutum*	CMF
Gohir.D13G062900.4.p	D13	8988121..8993084	*Gossypium hirsutum*	CMF
Gohir.D13G062900.6.p	D13	8988121..8993084	*Gossypium hirsutum*	CMF
Gohir.D13G062900.7.p	D13	8988122..8993084	*Gossypium hirsutum*	CMF
Gohir.D13G062900.8.p	D13	8988121..8993084	*Gossypium hirsutum*	CMF
Gohir.D13G062900.9.p	D13	8988122..8993084	*Gossypium hirsutum*	CMF
Gohir.D11G105700.3.p	D11	9492356..9497389	*Gossypium hirsutum*	PRR
Gohir.D11G105700.4.p	D11	9492357..9497389	*Gossypium hirsutum*	PRR
Gohir.D11G105700.6.p	D11	9492386..9497359	*Gossypium hirsutum*	PRR
Gohir.D11G105700.8.p	D11	9492386..9497359	*Gossypium hirsutum*	PRR
Gohir.D11G344400.1.p	D11	73108333..73119314	*Gossypium hirsutum*	PRR
Gohir.D11G344400.2.p	D11	91829330…91830127	*Gossypium hirsutum*	PRR
Gohir.D11G344400.3.p	D11	73108598..73119314	*Gossypium hirsutum*	PRR
Gohir.D11G344400.4.p	D11	73108598..73119314	*Gossypium hirsutum*	PRR
Gohir.D12G247100.1.p	D12	60486696..60489957	*Gossypium hirsutum*	PRR
Gohir.D12G247100.2.p	D12	60486696..60490005	*Gossypium hirsutum*	PRR
Gohir.D12G247100.3.p	D12	60486696..60490005	*Gossypium hirsutum*	PRR
Gohir.D12G247100.5.p	D12	60486696..60489957	*Gossypium hirsutum*	PRR
Gohir.A03G067700.1.p	A03	18501326..18506601	*Gossypium hirsutum*	PRR
Gohir.A03G067700.2.p	A03	18501326..18506601	*Gossypium hirsutum*	PRR
Gohir.A05G099900.5.p	A05	9258312..9264380	*Gossypium hirsutum*	PRR
Gohir.A05G099900.6.p	A05	9258312..9264380	*Gossypium hirsutum*	PRR

Gohir.A05G099900.7.p	A05	9259362..9264380	*Gossypium hirsutum*	PRR
Gohir.A05G099900.8.p	A05	9259362..9264380	*Gossypium hirsutum*	PRR
Gohir.A05G343600.1.p	A05	86030777..86037807	*Gossypium hirsutum*	PRR
Gohir.A05G343600.5.p	A05	86030774..86031896	*Gossypium hirsutum*	PRR
Gohir.A09G160600.2.p	A09	75266558..75273116	*Gossypium hirsutum*	PRR
Gohir.A09G160600.3.p	A09	75266558..75273116	*Gossypium hirsutum*	PRR
Gohir.A09G160600.4.p	A09	75266559..75273116	*Gossypium hirsutum*	PRR
Gohir.A11G016500.1.p	A11	1509664..1513507	*Gossypium hirsutum*	PRR
Gohir.A11G016500.2.p	A11	1509664..1513507	*Gossypium hirsutum*	PRR
Gohir.A11G016500.3.p	A11	1509664..1513507	*Gossypium hirsutum*	PRR
Gohir.A11G016500.4.p	A11	1509664..1513507	*Gossypium hirsutum*	PRR
Gohir.A11G101100.1.p	A11	10371699..10376656	*Gossypium hirsutum*	PRR
Gohir.A11G101100.2.p	A11	10371699..10376656	*Gossypium hirsutum*	PRR
Gohir.A11G101100.3.p	A11	10371987..10376465	*Gossypium hirsutum*	PRR
Gohir.A11G101100.4.p	A11	10371699..10376656	*Gossypium hirsutum*	PRR
Gohir.A11G101100.5.p	A11	10371987..10376465	*Gossypium hirsutum*	PRR
Gohir.A11G101100.6.p	A11	10371699..10376656	*Gossypium hirsutum*	PRR
Gohir.A11G325401.1.p	A11	122092990..122099189	*Gossypium hirsutum*	PRR
Gohir.A11G325401.2.p	A11	122092878..122099578	*Gossypium hirsutum*	PRR
Gohir.A11G325401.3.p	A11	122092989..122099578	*Gossypium hirsutum*	PRR
Gohir.A11G325401.4.p	A11	122092990..122099578	*Gossypium hirsutum*	PRR
Gohir.A12G246400.1.p	A12	105344012..105347598	*Gossypium hirsutum*	PRR
Gohir.A12G246400.4.p	A12	105344012..105347598	*Gossypium hirsutum*	PRR
Gohir.A12G246400.5.p	A12	105344052..105346730	*Gossypium hirsutum*	PRR
Gohir.D03G094200.1.p	D03	38165652..38170875	*Gossypium hirsutum*	PRR
Gohir.D04G000100.1.p	D04	174675..179169	*Gossypium hirsutum*	PRR
Gohir.D04G000100.2.p	D04	174675..179169	*Gossypium hirsutum*	PRR
Gohir.D04G000100.3.p	D04	174675..179169	*Gossypium hirsutum*	PRR
Gohir.D04G000100.4.p	D04	174675..179169	*Gossypium hirsutum*	PRR
Gohir.D04G082000.1.p	D04	17732712..17739443	*Gossypium hirsutum*	PRR
Gohir.D04G082000.2.p	D04	17732712..17739443	*Gossypium hirsutum*	PRR
Gohir.D05G099700.1.p	D05	8246212..8251561	*Gossypium hirsutum*	PRR
Gohir.D05G099700.10.p	D05	8245540..8250510	*Gossypium hirsutum*	PRR
Gohir.D05G099700.11.p	D05	8245539..8251561	*Gossypium hirsutum*	PRR
Gohir.A03G073100.1.p	A03	21025172..21026691	*Gossypium hirsutum*	COL
Gohir.A05G062400.1.p	A05	5953799..5955435	*Gossypium hirsutum*	COL
Gohir.A07G034300.1.p	A07	4233047..4234415	*Gossypium hirsutum*	COL
Gohir.A08G022000.1.p	A08	2689664..2691116	*Gossypium hirsutum*	COL
Gohir.A08G086800.1.p	A08	36191787..36194011	*Gossypium hirsutum*	COL
Gohir.A08G094500.1.p	A08	56261645..56263713	*Gossypium hirsutum*	COL
Gohir.A09G047500.1.p	A09	43786968..43788786	*Gossypium hirsutum*	COL
Gohir.A12G008800.11.p	A12	1053721..1056629	*Gossypium hirsutum*	COL
Gohir.A12G008800.12.p	A12	1053721…1056629	*Gossypium hirsutum*	COL
Gohir.A12G008800.2.p	A12	1053721..1056629	*Gossypium hirsutum*	COL

Gohir.A12G057200.1.p	A12	13039672..13045167	*Gossypium hirsutum*	COL
Gohir.A13G178800.1.p	A13	105383038..105384483	*Gossypium hirsutum*	COL
Gohir.A13G211600.1.p	A13	109166587..109168585	*Gossypium hirsutum*	COL
Gohir.D01G176000.1.p	D01	59520599..59522229	*Gossypium hirsutum*	COL
Gohir.D03G099600.1.p	D03	40004458..40008965	*Gossypium hirsutum*	COL
Gohir.D03G099600.2.p	D03	39946423..39948387	*Gossypium hirsutum*	COL
Gohir.D05G064900.1.p	D05	5154332..5155977	*Gossypium hirsutum*	COL
Gohir.D07G037800.1.p	D07	4000641..4002431	*Gossypium hirsutum*	COL
Gohir.D08G032600.1.p	D08	2568626..2570070	*Gossypium hirsutum*	COL
Gohir.D08G096200.1.p	D08	19930912..19932755	*Gossypium hirsutum*	COL
Gohir.D08G105900.1.p	D08	29965679..29967995	*Gossypium hirsutum*	COL
Gohir.D09G048500.1.p	D09	25211400..25213401	*Gossypium hirsutum*	COL
Gohir.D12G056900.1.p	D12	9968735..9970579	*Gossypium hirsutum*	COL
Gohir.D13G186900.1.p	D13	58781989..58783913	*Gossypium hirsutum*	COL
Gohir.D13G215600.1.p	D13	62588887..62591053	*Gossypium hirsutum*	COL
Gohir.A01G185300.1.p	A01	96358261..96359630	*Gossypium hirsutum*	COL
HORVU2Hr1G013400.32	2H	29123785..29127889	*Hordeum vulgare*	PRR
HORVU2Hr1G013400.34	2H	29124249..29127457	*Hordeum vulgare*	PRR
HORVU4Hr1G057550.2	4H	482660376..482666037	*Hordeum vulgare*	PRR
HORVU4Hr1G057550.3	4H	482660383..482665806	*Hordeum vulgare*	PRR
HORVU4Hr1G057550.4	4H	482660391..482666038	*Hordeum vulgare*	PRR
HORVU5Hr1G081620.1	5H	565156283..565160153	*Hordeum vulgare*	PRR
HORVU6Hr1G057630.1	6H	374866561..374869556	*Hordeum vulgare*	PRR
HORVU4Hr1G007420.1	4H	19477279..19479643	*Hordeum vulgare*	COL
HORVU4Hr1G007420.3	4H	19477293..19478703	*Hordeum vulgare*	COL
HORVU4Hr1G007420.4	4H	19477293..19478703	*Hordeum vulgare*	COL
HORVU5Hr1G051230.1	5H	399885632..399887538	*Hordeum vulgare*	COL
HORVU6Hr1G030080.4	6H	124453763..124455026	*Hordeum vulgare*	COL
HORVU6Hr1G030080.5	6H	124453763..124455035	*Hordeum vulgare*	COL
HORVU6Hr1G056000.2	6H	357491041..357492205	*Hordeum vulgare*	COL
HORVU6Hr1G072620.8	6H	504461571..504463292	*Hordeum vulgare*	COL
HORVU6Hr1G072620.9	6H	504461571..504463507	*Hordeum vulgare*	COL
HORVU6Hr1G073170.5	6H	507545415..507546806	*Hordeum vulgare*	COL
HORVU6Hr1G073170.6	6H	507545420..507546814	*Hordeum vulgare*	COL
HORVU6Hr1G073170.7	6H	507545430..507546806	*Hordeum vulgare*	COL
HORVU7Hr1G027560.1	7H	49216298..49217570	*Hordeum vulgare*	COL
HORVU5Hr1G081620.4	5H	565156800..565159905	*Hordeum vulgare*	PRR
Mapoly0085s0081.1.p	scaffold_85	653430…660786	*Marchantia polymorpha*	PRR
Mapoly0049s0067.1.p	scaffold_49	645333..647941	*Marchantia polymorpha*	COL
Mapoly0110s0028.1.p	scaffold_110	385628..390041	*Marchantia polymorpha*	COL
Mapoly0122s0029.1.p	scaffold_122	257934..260280	*Marchantia polymorpha*	COL
Mapoly0085s0081.2.p	scaffold_85	653444..660786	*Marchantia polymorpha*	PRR
Mapoly0085s0081.3.p	scaffold_85	653444..660786	*Marchantia polymorpha*	PRR
Mapoly0122s0007.1.p	scaffold_122	93724..101479	*Marchantia polymorpha*	PRR

Mapoly0122s0007.2.p	scaffold_122	95201..101479	*Marchantia polymorpha*	PRR
Mapoly0122s0007.3.p	scaffold_122	95201..101479	*Marchantia polymorpha*	PRR
GKY94971.1	scaffold_7	66948..67757	*Mayamaea pseudoterrestris*	PRR
Medtr1g067110.1	1	28871221…28881239	*Medicago truncatula*	PRR
Medtr1g067110.2	1	28871221..28881239	*Medicago truncatula*	PRR
Medtr1g067110.4	1	28871221..28881239	*Medicago truncatula*	PRR
Medtr1g067110.6	1	28871221..28881239	*Medicago truncatula*	PRR
Medtr1g067110.7	1	28871221..28881239	*Medicago truncatula*	PRR
Medtr1g067110.8	1	13728441..13734998	*Medicago truncatula*	PRR
Medtr1g013450.1	1	3419850..3420903	*Medicago truncatula*	COL
Medtr1g110870.1	1	50054285..50056406	*Medicago truncatula*	COL
Medtr3g105710.1	3	48758302..48759914	*Medicago truncatula*	COL
Medtr4g128930.1	4	53656301..53658369	*Medicago truncatula*	COL
Medtr7g018170.1	7	5833561..5837191	*Medicago truncatula*	COL
Medtr8g104190.1	8	43867638..43869522	*Medicago truncatula*	COL
Medtr3g037390.1	3	13728441..13734998	*Medicago truncatula*	PRR
Medtr3g037390.2	3	42411882..42416036	*Medicago truncatula*	PRR
Medtr3g092780.1	3	42411881..42416018	*Medicago truncatula*	PRR
Medtr3g092780.2	3	42411881..42416018	*Medicago truncatula*	PRR
Medtr3g092780.3	3	42411881..42416018	*Medicago truncatula*	PRR
Medtr3g092780.4	3	42411907..42416036	*Medicago truncatula*	PRR
Medtr4g061360.1	4	22682971..22690792	*Medicago truncatula*	PRR
Medtr4g061360.2	4	22682971..22690792	*Medicago truncatula*	PRR
Medtr4g061360.3	4	22682971..22690792	*Medicago truncatula*	PRR
Medtr4g061360.4	4	22682971..22690791	*Medicago truncatula*	PRR
Medtr4g061360.5	4	45127441..45132453	*Medicago truncatula*	PRR
Medtr4g108880.1	4	45127441..45132438	*Medicago truncatula*	PRR
Medtr4g108880.2	4	49074826..49076181	*Medicago truncatula*	PRR
Medtr1g044785.1	4	16921077..16923253	*Medicago truncatula*	CMF
Medtr1g073350.1	7	32553600..32555963	*Medicago truncatula*	CMF
Medtr2g088900.1	8	37490665..37495477	*Medicago truncatula*	CMF
Medtr7g118260.1	7	49081893..49086030	*Medicago truncatula*	PRR
Medtr7g118260.2	7	49081784..49086030	*Medicago truncatula*	PRR
Medtr8g024260.1	8	8895233..8900931	*Medicago truncatula*	PRR
KAG7349401.1	tig00000071	1886873..1888801	*Nitzschia inconspicua*	PRR
KAG7351233.1	tig00000074	3043991..3045964	*Nitzschia inconspicua*	PRR
KAG7354330.1	tig00000012	2409502..2410540	*Nitzschia inconspicua*	PRR
KAG7374414.1	tig00000111	1826068..1826445	*Nitzschia inconspicua*	PRR
LOC_Os02g08150.1	2	4315275..4316961	*Oryza sativa*	COL
LOC_Os02g39710.1	2	23989750..23991293	*Oryza sativa*	COL
LOC_Os02g49880.1	2	30472788..30475773	*Oryza sativa*	COL
LOC_Os03g50310.1	3	28686958..28689716	*Oryza sativa*	COL
LOC_Os04g42020.1	4	24889983..24891487	*Oryza sativa*	COL
LOC_Os06g15330.1	6	8704831..8706949	*Oryza sativa*	COL

LOC_Os06g44450.1	6	26843087..26844776	*Oryza sativa*	COL
LOC_Os02g40510.1	2	24569294..24572560	*Oryza sativa*	PRR
LOC_Os03g17570.1	3	9759479..9768690	*Oryza sativa*	PRR
LOC_Os03g17570.2	3	9759479..9768690	*Oryza sativa*	PRR
LOC_Os03g17570.3	3	9759479..9768656	*Oryza sativa*	PRR
LOC_Os07g49460.1	7	29616705..29629223	*Oryza sativa*	PRR
LOC_Os07g49460.2	7	29616732..29629223	*Oryza sativa*	PRR
LOC_Os07g49460.3	7	29616705..29629220	*Oryza sativa*	PRR
LOC_Os07g49460.4	7	29616705..29629220	*Oryza sativa*	PRR
LOC_Os09g36220.1	9	20885172..20889843	*Oryza sativa*	PRR
LOC_Os09g36220.2	9	20885172..20889843	*Oryza sativa*	PRR
LOC_Os11g05930.1	11	2789002…2793735	*Oryza sativa*	PRR
LOC_Os11g05930.2	11	2788996..2793735	*Oryza sativa*	PRR
LOC_Os11g05930.3	11	2789002..2793735	*Oryza sativa*	PRR
LOC_Os11g05930.4	11	2789002…2793728	*Oryza sativa*	PRR
EEC44655.1	21	397399..400687	*Phaeodactylum tricornutum* CCAP 1055/1	PRR
XP_002183986.1	21	684..2258	*Phaeodactylum tricornutum* CCAP 1055/1	PRR
Potri.002G179800.1.p	2	14117553..14123267	*Populus trichocarpa*	PRR
Potri.008G171500.1.p	8	11846355..11848970	*Populus trichocarpa*	CMF
Potri.008G171500.2.p	8	11846355..11850603	*Populus trichocarpa*	CMF
Potri.010G066100.2.p	10	9460824..9465538	*Populus trichocarpa*	CMF
Potri.010G066100.3.p	10	9462575..9465197	*Populus trichocarpa*	CMF
Potri.010G066100.4.p	10	9460830..9465539	*Populus trichocarpa*	CMF
Potri.002G179800.15.p	2	14117543..14123501	*Populus trichocarpa*	PRR
Potri.004G108320.1.p	4	9572140..9574211	*Populus trichocarpa*	COL
Potri.006G173600.1.p	6	17514652..17517264	*Populus trichocarpa*	COL
Potri.006G267700.1.p	6	26229913..26232733	*Populus trichocarpa*	COL
Potri.008G120400.3.p	8	7794617..7797846	*Populus trichocarpa*	COL
Potri.010G125100.1.p	10	14244777..14247095	*Populus trichocarpa*	COL
Potri.014G134601.2.p	14	9020685..9028372	*Populus trichocarpa*	COL
Potri.015G054600.1.p	15	7464225..7466727	*Populus trichocarpa*	COL
Potri.017G107500.3.p	17	11638205..11640308	*Populus trichocarpa*	COL
Potri.018G013800.1.p	18	923840..926502	*Populus trichocarpa*	COL
Potri.018G096084.1.p	18	11697477..11700055	*Populus trichocarpa*	COL
Potri.002G179800.2.p	2	14117609..14123266	*Populus trichocarpa*	PRR
Potri.002G179800.9.p	2	14117543..14123501	*Populus trichocarpa*	PRR
Potri.008G046200.3.p	8	2674335..2683096	*Populus trichocarpa*	PRR
Potri.008G046200.9.p	8	2674339..2682947	*Populus trichocarpa*	PRR
Potri.010G215200.14.p	10	20262808..20271048	*Populus trichocarpa*	PRR
Potri.010G215200.23.p	10	20262813..20271048	*Populus trichocarpa*	PRR
Potri.010G215200.24.p	10	20262809..20270989	*Populus trichocarpa*	PRR
Potri.010G215200.25.p	10	20262812..20270989	*Populus trichocarpa*	PRR

Potri.010G215200.26.p	10	20262809..20270988	*Populus trichocarpa*	PRR
Potri.012G005900.11.p	12	173794..180376	*Populus trichocarpa*	PRR
Potri.012G005900.12.p	12	174695..180108	*Populus trichocarpa*	PRR
Potri.014G106000.13.p	19	7107684…7112749	*Populus trichocarpa*	PRR
Potri.014G106000.14.p	19	7107691..7112749	*Populus trichocarpa*	PRR
Potri.014G106000.15.p	19	7107701..7112847	*Populus trichocarpa*	PRR
Potri.014G106000.16.p	19	7107696..7112819	*Populus trichocarpa*	PRR
Potri.014G106000.17.p	3	7107691..7112802	*Populus trichocarpa*	PRR
Potri.014G106000.18.p	3	7107754…7112746	*Populus trichocarpa*	PRR
Potri.015G002300.2.p	Chr15	138231..142709	*Populus trichocarpa*	PRR
Potri.015G002300.3.p	Chr15	112482..116960	*Populus trichocarpa*	PRR
Potri.015G002300.4.p	Chr15	112482..116960	*Populus trichocarpa*	PRR
Potri.015G061900.5.p	Chr15	8461838..8467297	*Populus trichocarpa*	PRR
CAB9513243.1	Sro_contig580	5665..6513	*Seminavis robusta*	PRR
CAB9508819.1	Sro_contig362	12213..14248	*Seminavis robusta*	PRR
Solyc02g089500.4.1	2	49351829..49352535	*Solanum lycopersicum*	COL
Solyc02g089520.2.1	2	49357153..49358925	*Solanum lycopersicum*	COL
Solyc02g089540.3.1	2	49365916..49368618	*Solanum lycopersicum*	COL
Solyc09g090650.3.1	9	66199248..66202425	*Solanum lycopersicum*	CMF
Solyc12g096940.3.1	12	65167779..65169533	*Solanum lycopersicum*	CMF
Solyc03g119540.3.1	3	62565853..62567474	*Solanum lycopersicum*	COL
Solyc04g007210.3.1	4	940495..942888	*Solanum lycopersicum*	COL
Solyc05g009310.3.1	5	3490063..3492184	*Solanum lycopersicum*	COL
Solyc07g006630.4.1	7	1468325..1470183	*Solanum lycopersicum*	COL
Solyc08g006530.4.1	8	1154652..1156466	*Solanum lycopersicum*	COL
Solyc12g096500.2.1	12	64913638..64915137	*Solanum lycopersicum*	COL
Solyc03g081240.3.1	3	46704175..46709293	*Solanum lycopersicum*	PRR
Solyc03g081270.2.1	3	46723777..46726320	*Solanum lycopersicum*	PRR
Solyc03g115770.3.1	3	59800548..59806781	*Solanum lycopersicum*	PRR
Solyc04g049670.4.1	4	42073726..42080420	*Solanum lycopersicum*	PRR
Solyc06g069690.4.1	5	41034051..41040009	*Solanum lycopersicum*	PRR
Solyc10g005030.4.1	10	64712891..64719450	*Solanum lycopersicum*	PRR
Sobic.001G411400.1.p	1	73571519..73578856	*Sorghum bicolor*	PRR
Sobic.001G118100.1.p	1	9316865..9322191	*Sorghum bicolor*	COL
Sobic.004G211200.1.p	4	58610987..58612663	*Sorghum bicolor*	COL
Sobic.004G249500.1.p	4	62159984..62162412	*Sorghum bicolor*	COL
Sobic.004G252300.1.p	4	62357099..62359638	*Sorghum bicolor*	COL
Sobic.004G252300.2.p	4	62357099..62359297	*Sorghum bicolor*	COL
Sobic.006G135100.1.p	6	51219318..51221049	*Sorghum bicolor*	COL
Sobic.010G108500.1.p	10	10905117..10907343	*Sorghum bicolor*	COL
Sobic.010G115800.1.p	10	12485039..12486838	*Sorghum bicolor*	COL
Sobic.010G214000.1.p	10	57279824..57281835	*Sorghum bicolor*	COL
Sobic.001G411400.2.p	1	73571676..73578611	*Sorghum bicolor*	PRR
Sobic.002G275100.1.p	2	67081482..67087636	*Sorghum bicolor*	PRR

Sobic.002G275100.2.p	2	67081486..67087046	*Sorghum bicolor*	PRR
Sobic.002G275100.3.p	2	67081763..67087039	*Sorghum bicolor*	PRR
Sobic.004G216700.1.p	4	59135641..59139159	*Sorghum bicolor*	PRR
Sobic.005G044400.1.p	5	4250186..4255684	*Sorghum bicolor*	PRR
Sobic.006G057933.1.p	6	41669408..41671712	*Sorghum bicolor*	PRR
Sobic.006G057933.2.p	6	41669544..41671664	*Sorghum bicolor*	PRR
Sphmag01G165500.1.p	LG01	27178239…27186832	*Sphagnum magellanicum*	PRR
Sphmag06G078700.1.p	06G	14110040..14115168	*Sphagnum magellanicum*	COL
Sphmag16G095000.1.p	16G	16957734..16960816	*Sphagnum magellanicum*	COL
Sphmag17G097600.1.p	17G	17738512..17743218	*Sphagnum magellanicum*	COL
Sphmag19G087400.1.p	19G	15631411..15634742	*Sphagnum magellanicum*	COL
Sphmag01G165500.2.p	LG01	27178239..27186832	*Sphagnum magellanicum*	PRR
Sphmag01G165500.3.p	LG01	27178239..27186572	*Sphagnum magellanicum*	PRR
Sphmag01G165500.4.p	LG01	27178239..27186572	*Sphagnum magellanicum*	PRR
Sphmag02G009400.1.p	LG02	1450846..1458012	*Sphagnum magellanicum*	PRR
Sphmag02G009400.2.p	LG02	1450846..1458053	*Sphagnum magellanicum*	PRR
Sphmag02G009400.3.p	LG02	1450846..1458053	*Sphagnum magellanicum*	PRR
Sphmag02G009400.4.p	LG02	1450846..1458053	*Sphagnum magellanicum*	PRR
Sphmag02G009400.5.p	LG02	1450846.146	*Sphagnum magellanicum*	PRR
Sphmag02G009400.6.p	LG02	1450846..1458012	*Sphagnum magellanicum*	PRR
Sphmag04G059300.1.p	16	9503289..9508628	*Sphagnum magellanicum*	PRR
Sphmag10G024100.1.p	11	4093177..4100688	*Sphagnum magellanicum*	PRR
Sphmag15G081400.1.p	10	13946684..13952172	*Sphagnum magellanicum*	PRR
Thecc.01G333100.1.p	1	35463519..35468475	*Theobroma cacao*	PRR
Thecc.01G333100.2.p	1	35463564..35468332	*Theobroma cacao*	PRR
Thecc.02G231000.1.p	2	29147939..29149627	*Theobroma cacao*	COL
Thecc.03G034600.1.p	3	2436803..2438867	*Theobroma cacao*	COL
Thecc.03G112600.1.p	3	23523848..23526836	*Theobroma cacao*	COL
Thecc.03G112600.2.p	3	23523848..23526836	*Theobroma cacao*	COL
Thecc.04G263300.1.p	4	32253283..32257555	*Theobroma cacao*	CMF
Thecc.04G263300.2.p	4	32253283..32256876	*Theobroma cacao*	CMF
Thecc.04G113300.1.p	4	21069605..21072385	*Theobroma cacao*	COL
Thecc.09G011100.1.p	9	578463..580016	*Theobroma cacao*	COL
Thecc.09G165200.1.p	9	11151754..11153758	*Theobroma cacao*	COL
Thecc.03G093700.1.p	1	20933624..20940322	*Theobroma cacao*	PRR
Thecc.03G093700.2.p	1	20933624..20940273	*Theobroma cacao*	PRR
Thecc.03G093700.3.p	1	20935098..20940273	*Theobroma cacao*	PRR
Thecc.03G208800.1.p	3	29321514..29326367	*Theobroma cacao*	PRR
Thecc.03G208800.2.p	3	29321339..29326915	*Theobroma cacao*	PRR
Thecc.05G051700.1.p	5	2440119..2446619	*Theobroma cacao*	PRR
Thecc.05G051700.2.p	5	2440119..2446621	*Theobroma cacao*	PRR
Thecc.05G051700.3.p	5	2440119..2446621	*Theobroma cacao*	PRR
Thecc.05G051700.4.p	5	2440119..2446621	*Theobroma cacao*	PRR
Thecc.05G051700.5.p	5	2440119..2446609	*Theobroma cacao*	PRR

Thecc.05G051700.6.p	5	2440119..2446609	*Theobroma cacao*	PRR
Thecc.05G051700.7.p	5	2440119..2445742	*Theobroma cacao*	PRR
Thecc.09G335000.1.p	9	40750419..40756246	*Theobroma cacao*	PRR
Thecc.10G064700.1.p	10	3633889..3648694	*Theobroma cacao*	PRR
Thecc.10G064700.2.p	10	3633798..3648646	*Theobroma cacao*	PRR
Thecc.10G064700.3.p	10	3633889..3648694	*Theobroma cacao*	PRR
Traes_4BS_46930D906.1	4BS	27276..28886	*Triticum aestivum*	COL
Traes_4DS_50A614BFB.1	4DS	2..1784	*Triticum aestivum*	COL
Traes_5BL_DB6E9FDB9.2	5BL	8351..10845	*Triticum aestivum*	COL
Traes_6DL_9CC066885.1	6DL	15..1581	*Triticum aestivum*	COL
Traes_6DL_9CC066885.2	6DL	2..1581	*Triticum aestivum*	COL
Traes_4AS_B2CDBFB54.2	4AS	8534..13141	*Triticum aestivum*	PRR
Traes_4AS_B2CDBFB54.2	4AS	8534..13141	*Triticum aestivum*	PRR
Traes_4AS_B2CDBFB54.3	4AS	6939..13172	*Triticum aestivum*	PRR
Traes_4AS_B2CDBFB54.4	4AS	8335..13208	*Triticum aestivum*	PRR
Traes_4AS_B2CDBFB54.8	4AS	8348..13169	*Triticum aestivum*	PRR
Traes_4AS_B2CDBFB54.9	4AS	8483..13169	*Triticum aestivum*	PRR
Traes_4BL_430501C10.1	4BL	5643..10966	*Triticum aestivum*	PRR
Traes_4BL_430501C10.2	4BL	5629..11272	*Triticum aestivum*	PRR
Traes_4BS_4BCA03A6C.1	4BS	4870..11422	*Triticum aestivum*	PRR
Traes_4DL_EE41726EA.1	4DL	2133..7846	*Triticum aestivum*	PRR
Traes_4DL_EE41726EA.2	4DL	2125..7831	*Triticum aestivum*	PRR
Traes_4DL_EE41726EA.3	4DL	2179..7828	*Triticum aestivum*	PRR
Traes_4DL_EE41726EA.4	4DL	2206..7831	*Triticum aestivum*	PRR
Traes_4DL_EE41726EA.5	4DL	2163..7831	*Triticum aestivum*	PRR
Traes_4DS_A8B6C23C4.1	4DS	4578..11187	*Triticum aestivum*	PRR
Traes_5AL_852A1474C.1	5AL	1863..5488	*Triticum aestivum*	PRR
Traes_5DL_8CE2482E6.1	5DL	282..3189	*Triticum aestivum*	PRR
Traes_5DL_8CE2482E6.2	5DL	49..3536	*Triticum aestivum*	PRR
Traes_5DL_8CE2482E6.3	5DL	49..3536	*Triticum aestivum*	PRR
Traes_6AL_A0A31AA9F.1	6AL	942..3821	*Triticum aestivum*	PRR
Traes_6BL_ED40C8806.1	6BL	72..2820	*Triticum aestivum*	PRR
Traes_6DL_C215BACFD.1	6DL	331..6202	*Triticum aestivum*	PRR
Traes_2AS_2FCD59730.1	2AS	92..5687	*Triticum aestivum*	PRR
Traes_2AS_2FCD59730.2	2AS	98..5686	*Triticum aestivum*	PRR
Traes_4AL_72C3E1EAD.1	4AL	3441..9275	*Triticum aestivum*	PRR
Traes_4AL_72C3E1EAD.2	4AL	1595..9465	*Triticum aestivum*	PRR
Traes_4AL_72C3E1EAD.3	4AL	1572..9192	*Triticum aestivum*	PRR
Traes_4AS_B2CDBFB54.1	4AS	7745..12890	*Triticum aestivum*	PRR
Traes_4AS_B2CDBFB54.10	4AS	8581..13141	*Triticum aestivum*	PRR
Traes_4AS_B2CDBFB54.11	4AS	6956..13208	*Triticum aestivum*	PRR
Traes_4AS_B2CDBFB54.12	4AS	7013..13169	*Triticum aestivum*	PRR
Traes_4AS_B2CDBFB54.13	4AS	8337..13169	*Triticum aestivum*	PRR
Traes_4AS_B2CDBFB54.14	4AS	8337..13169	*Triticum aestivum*	PRR

VIT_201s0011g03520.1	1	3190707..3192646	*Vitis vinifera*	COL
VIT_201s0011g03520.2	1	3190849..3192646	*Vitis vinifera*	COL
VIT_205s0049g01830.1	5	9258167..9260111	*Vitis vinifera*	CMF
VIT_204s0008g07340.1	4	7669873..7671263	*Vitis vinifera*	COL
VIT_211s0052g01800.1	11	19620989..19622158	*Vitis vinifera*	COL
VIT_214s0083g00640.1	14	22696209..22698176	*Vitis vinifera*	COL
VIT_214s0083g00640.2	14	22696209..22697243	*Vitis vinifera*	COL
VIT_206s0004g03650.1	6	4607415..4629645	*Vitis vinifera*	PRR
VIT_206s0004g03650.2	6	4607415..4629796	*Vitis vinifera*	PRR
VIT_206s0004g03650.3	6	4613319..4629796	*Vitis vinifera*	PRR
VIT_206s0004g03650.4	6	4607415..4629645	*Vitis vinifera*	PRR
VIT_213s0067g03390.1	13	1859685..1891150	*Vitis vinifera*	PRR
VIT_213s0067g03390.2	13	1876876..1891150	*Vitis vinifera*	PRR
VIT_213s0067g03390.3	13	1889097..1891150	*Vitis vinifera*	PRR
VIT_215s0048g02540.1	15	16686733..16691695	*Vitis vinifera*	PRR
VIT_215s0048g02540.2	15	16686733..16691695	*Vitis vinifera*	PRR
VIT_215s0048g02540.3	15	16686733..16691695	*Vitis vinifera*	PRR
VIT_215s0048g02540.5	15	16686733..16691695	*Vitis vinifera*	PRR
VIT_215s0048g02540.6	15	16686733..16690627	*Vitis vinifera*	PRR
VIT_216s0098g00900.1	16	21231705..21237766	*Vitis vinifera*	PRR
VIT_216s0098g00900.2	16	21231705..21237766	*Vitis vinifera*	PRR
VIT_216s0098g00900.3	16	21231705..21234550	*Vitis vinifera*	PRR
VIT_217s0000g06570.1	17	7101672..7107338	*Vitis vinifera*	PRR
VIT_217s0000g06570.2	17	7101672..7107338	*Vitis vinifera*	PRR
VIT_217s0000g06570.3	17	7101672..7107338	*Vitis vinifera*	PRR
VIT_217s0000g06570.4	17	7101672..7107338	*Vitis vinifera*	PRR
VIT_217s0000g06570.5	17	7101672..7107338	*Vitis vinifera*	PRR
VIT_217s0000g06570.6	17	7101672..7107338	*Vitis vinifera*	PRR
Vocar.0036s0068.1.p	scaffold_36	546960..552203	*Volvox cartei*	PRR
Vocar.0074s0018.2.p	scaffold_74	133773..150013	*Volvox cartei*	PRR
Vocar.0074s0018.3.p	scaffold_74	1337731..36335	*Volvox cartei*	PRR
GRMZM2G012717_P01	4	38579200..38584266	*Zea mays*	COL
GRMZM2G021777_P01	4	4179652..2885473	*Zea mays*	COL
GRMZM2G038783_P01	7	11138091..11143177	*Zea mays*	COL
GRMZM2G041991_P01	3	17550832..17556959	*Zea mays*	COL
GRMZM2G095598_P01	2	4185541..4190036	*Zea mays*	COL
GRMZM2G107886_P01	8	4184806..4189950	*Zea mays*	COL
GRMZM2G114137_P01	8	48579300..48584466	*Zea mays*	COL
GRMZM2G144346_P01	3	2179652..2184473	*Zea mays*	COL
GRMZM2G148772_P01	3	2179652..2184519	*Zea mays*	COL
GRMZM2G005732_P03	3	19988196..19988358	*Zea mays*	PRR
GRMZM2G020081_P01	3	260195858..260198235	*Zea mays*	PRR
GRMZM2G033962_P01	4	72747838..72748728	*Zea mays*	PRR
GRMZM2G095727_P01	4	35964652..35967135	*Zea mays*	PRR

GRMZM2G095727_P05	4	35964652..35865145	*Zea mays*	PRR
GRMZM2G179024_P01	4	65109547..65109783	*Zea mays*	PRR
AC233888.1_FGP002	10	130457158..130458769	*Zea mays*	COL
GRMZM2G367834_P01	8	130926648..130938454	*Zea mays*	PRR

Table S2. Conserved motif sequence of all obtained CCT genes from 30 species

Name	Motif sequence
Motif1	LCKIMSHKTF KNIPVIMMSSHDSMGIVFKCLSKGAVDFLVKPIRKNELKNLWQHVWRRCH
Motif2	KFRQKRKERCFEKKVRYQSRKRLAEQRPR VRGQFVRQAV
Motif3	PLCEFCRVVRAVVYCKSDSARLCLHCDGCVHS
Motif4	LPRMVLRVLLVEADDSTRQII
Motif5	NIDLILTEVDLPSISGFALLTLIMEHKIC
Motif6	VKGQFVRQVQN
Motif7	RNILRHSDLSAFSRY NAASTANQAP
Motif8	SDNNTGSNDEDDNGSVGLNVQDGSDNGSGTQ
Motif9	LLRNCSYEVTAVANG
Motif10	KGGAGGGIGSGGRNVVDQNRFAQREAALN
Motif11	SLCQGCDWNPNDCSALGHRRVALNCYTGCPSLAEFSRIWSFVFDANSSLG
Motif12	AFSRYNSGST ANQAPTGNVGSCSPLDNSSEAVKTDSMKNFQSTSNSIPPKQ
Motif13	SWTKRAVEVDSHKPVSQWDQIAECPDST
Motif14	KFEFAPQLELSLKRLYPSSSK
Motif15	RGIQQQQQPPEHNDLSLKKMAADAPHCGSSNVLGGPVEGNAGNYSVNGSASGSNHGSNGP
Motif16	CDTCRSAACTIYCRADSAYLCAGCDARIH
Motif17	TCKADAAALCVTCDRDIHSANPLARRHER VPVVPFYDSA
Motif18	VGSNNIDMGSTTNNAFAKAAVDKNKSAASST
Motif19	SSCTKPDMEAESAHMDNMQEFSRMIQGKS
Motif20	DKLDEIESCVRYQPRMDQSQIIPSNPNYTPY
Motif21	DEIFYCSQVATRYHLEDGGMDCLLMDKNI
Motif22	SHQQVQVQHHHHHYHHYHHHVHSMQEQQQ
Motif23	LQAWKILEDLT
Motif24	PQGLVHSNMPLQFPNIVGENNSTEYQDCG

(A)

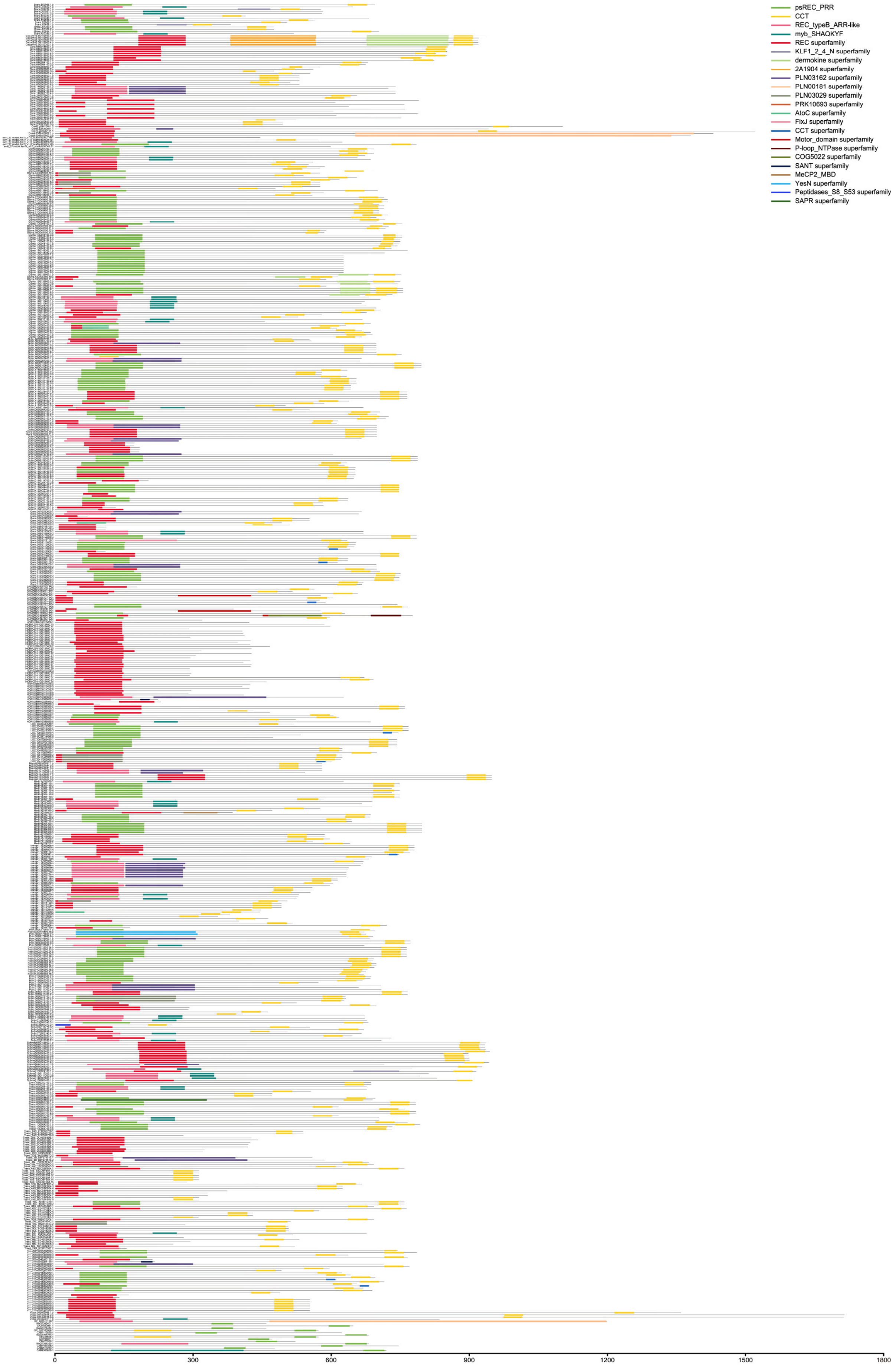
psREC_PRR
CCT
REC_typeB_ARR-like
myb_SHAQKYF
REC superfamily
KLF1_2_4_N superfamily
dermokine superfamily
2A1904 superfamily
PLN03162 superfamily
PLN00181 superfamily
PLN03029 superfamily
PRK10693 superfamily
AtoC superfamily
FixJ superfamily
CCT superfamily
Motor_domain superfamily
P-loop_NTPase superfamily
COG5022 superfamily
SANT superfamily
MeCP2_MBD
YesN superfamily
Peptidases_S8_S53 superfamily
SAPR superfamily
0
300
600
900
1200
1500
1800

(B)

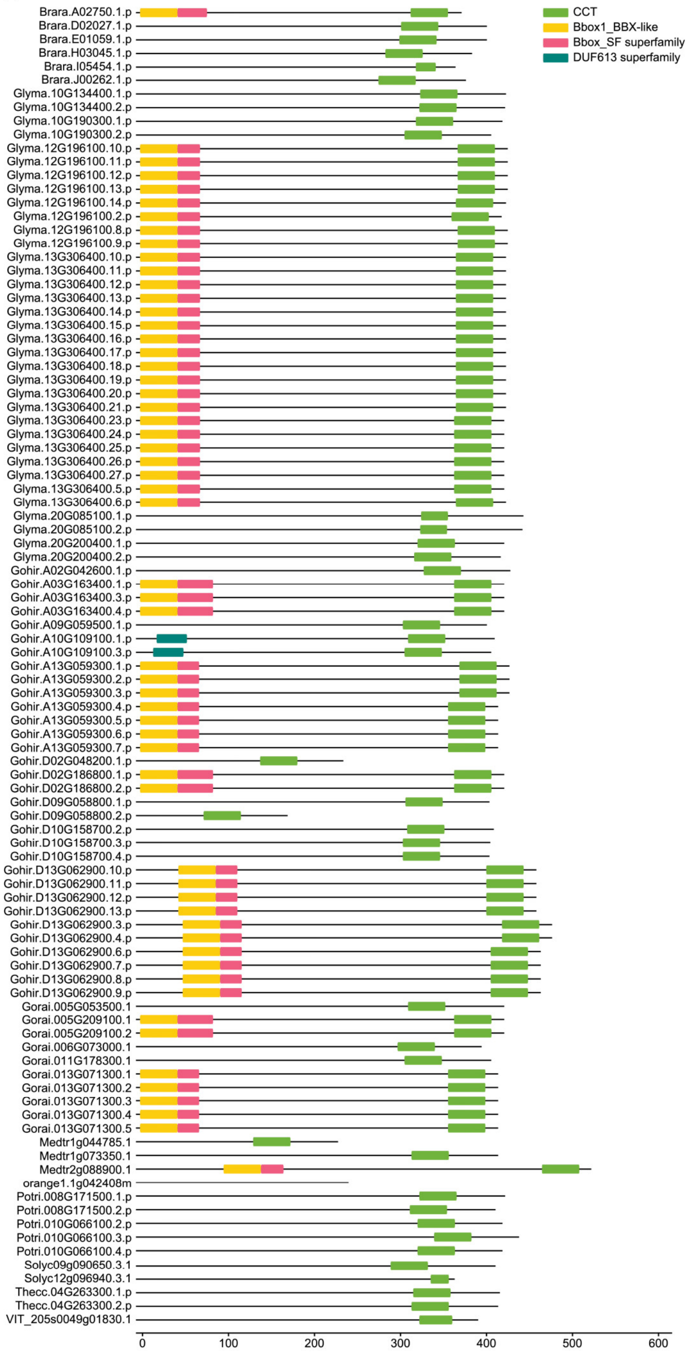

CCT
Bbox1_BBX-like
Bbox_SF superfamily
DUF613 superfamily
Brara.A02750.1.p
Brara.D02027.1.p
Brara.E01059.1.p
Brara.H03045.1.p
Brara.I05454.1.p
Brara.J00262.1.p
Glyma.10G134400.1.p
Glyma.10G134400.2.p
Glyma.10G190300.1.p
Glyma.10G190300.2.p
Glyma.12G196100.10.p
Glyma.12G196100.11.p
Glyma.12G196100.12.p
Glyma.12G196100.13.p
Glyma.12G196100.14.p
Glyma.12G196100.2.p
Glyma.12G196100.8.p
Glyma.12G196100.9.p
Glyma.13G306400.10.p
Glyma.13G306400.11.p
Glyma.13G306400.12.p
Glyma.13G306400.13.p
Glyma.13G306400.14.p
Glyma.13G306400.15.p
Glyma.13G306400.16.p
Glyma.13G306400.17.p
Glyma.13G306400.18.p
Glyma.13G306400.19.p
Glyma.13G306400.20.p
Glyma.13G306400.21.p
Glyma.13G306400.23.p
Glyma.13G306400.24.p
Glyma.13G306400.25.p
Glyma.13G306400.26.p
Glyma.13G306400.27.p
Glyma.13G306400.5.p
Glyma.13G306400.6.p
Glyma.20G085100.1.p
Glyma.20G085100.2.p
Glyma.20G200400.1.p
Glyma.20G200400.2.p
Gohir.A02G042600.1.p
Gohir.A03G163400.1.p
Gohir.A03G163400.3.p
Gohir.A03G163400.4.p
Gohir.A09G059500.1.p
Gohir.A10G109100.1.p
Gohir.A10G109100.3.p
Gohir.A13G059300.1.p
Gohir.A13G059300.2.p
Gohir.A13G059300.3.p
Gohir.A13G059300.4.p
Gohir.A13G059300.5.p
Gohir.A13G059300.6.p
Gohir.A13G059300.7.p
Gohir.D02G048200.1.p
Gohir.D02G186800.1.p
Gohir.D02G186800.2.p
Gohir.D09G058800.1.p
Gohir.D09G058800.2.p
Gohir.D10G158700.2.p
Gohir.D10G158700.3.p
Gohir.D10G158700.4.p
Gohir.D13G062900.10.p
Gohir.D13G062900.11.p
Gohir.D13G062900.12.p
Gohir.D13G062900.13.p
Gohir.D13G062900.3.p
Gohir.D13G062900.4.p
Gohir.D13G062900.6.p
Gohir.D13G062900.7.p
Gohir.D13G062900.8.p
Gohir.D13G062900.9.p
Gorai.005G053500.1
Gorai.005G209100.1
Gorai.005G209100.2
Gorai.006G073000.1
Gorai.011G178300.1
Gorai.013G071300.1
Gorai.013G071300.2
Gorai.013G071300.3
Gorai.013G071300.4
Gorai.013G071300.5
Medtr1g044785.1
Medtr1g073350.1
Medtr2g088900.1
orange1.1g042408m
Potri.008G171500.1.p
Potri.008G171500.2.p
Potri.010G066100.2.p
Potri.010G066100.3.p
Potri.010G066100.4.p
Solyc09g090650.3.1
Solyc12g096940.3.1
Thecc.04G263300.1.p
Thecc.04G263300.2.p
VIT_205s0049g01830.1
0
100
200
300
400
500
600

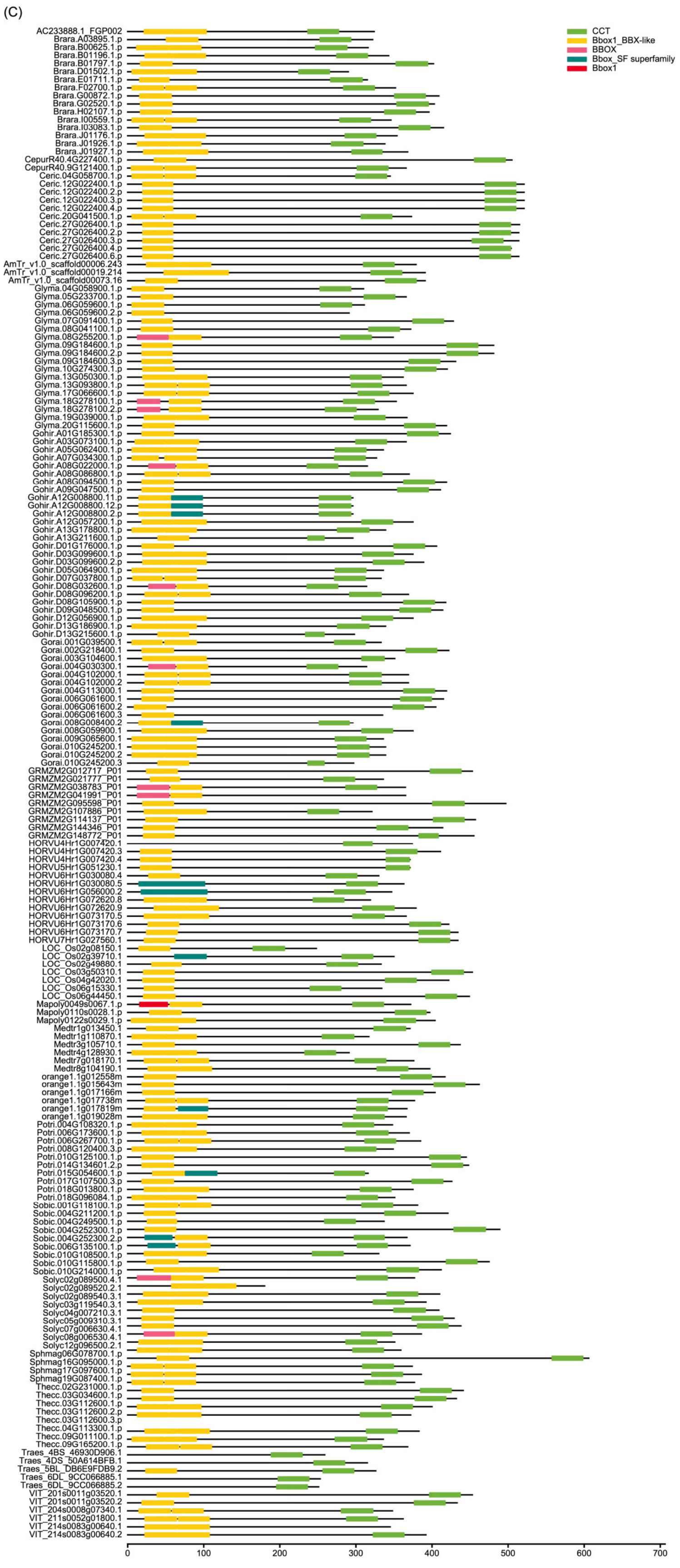

Figure. S1 Domains of the CCT proteins (A) Domains of the PRR proteins subfamily (B) Domains of the CMF proteins subfamily (C) Domains of the COL proteins subfamily.

(A)

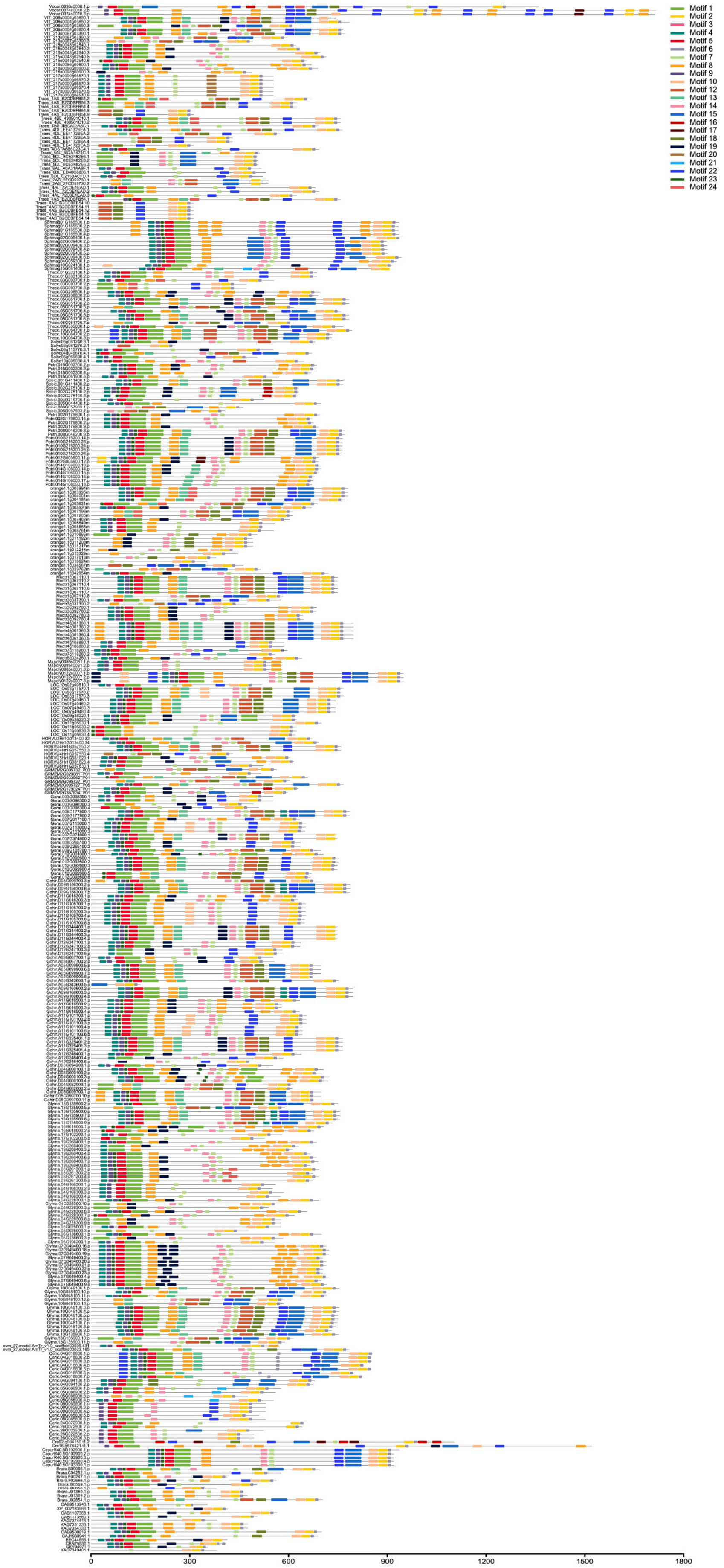
Motif 1
Motif 2
Motif 3
Motif 4
Motif 5
Motif 6
Motif 7
Motif 8
Motif 9
Motif 10
Motif 12
Motif 13
Motif 14
Motif 15
Motif 16
Motif 17
Motif 18
Motif 19
Motif 20
Motif 21
Motif 22
Motif 23
Motif 24
0
300
600
900
1200
1500
1800

(B)

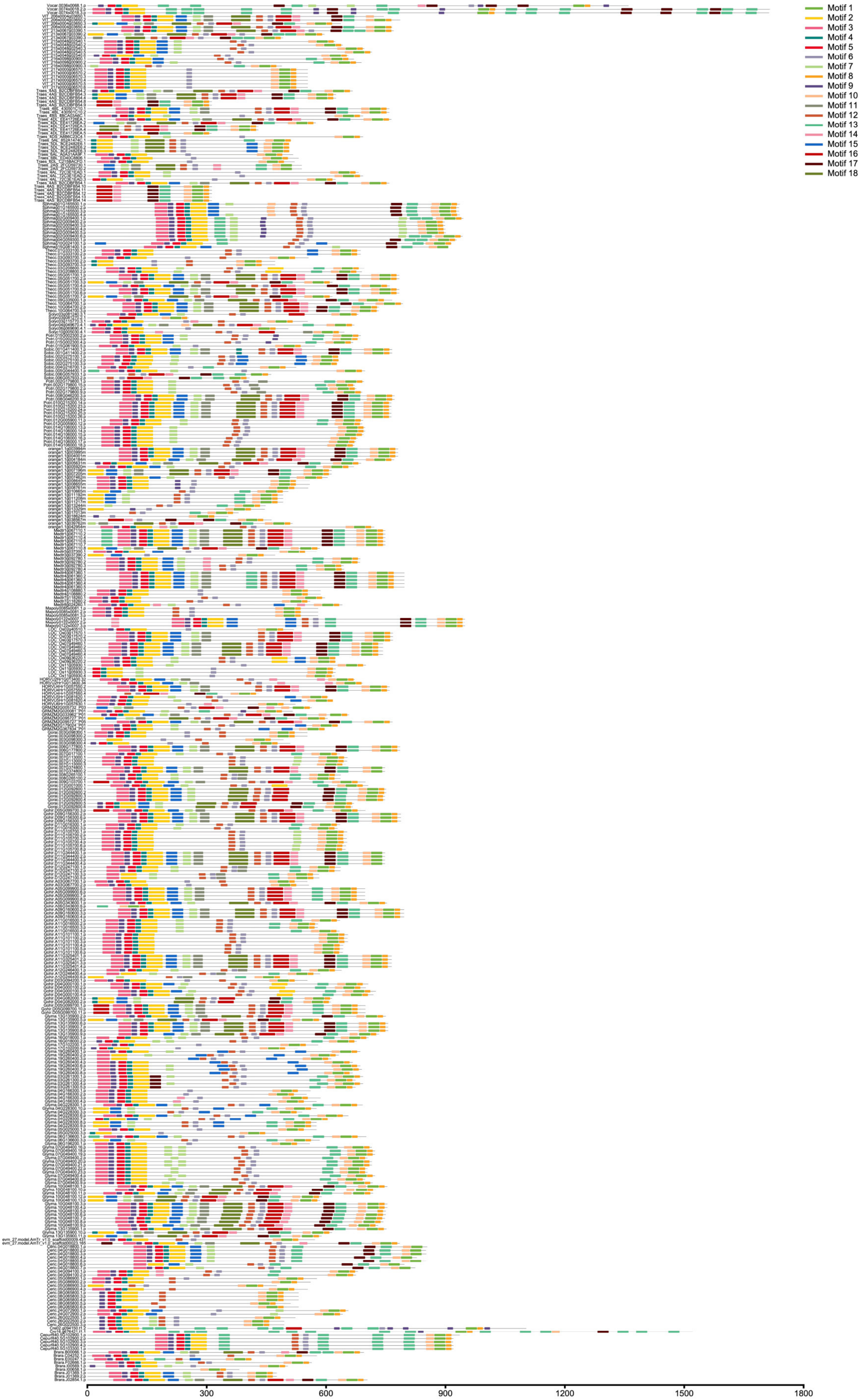
Motif 1
Motif 2
Motif 3
Motif 4
Motif 5
Motif 6
Motif 7
Motif 8
Motif 9
Motif 10
Motif 11
Motif 12
Motif 13
Motif 14
Motif 15
Motif 16
Motif 17
Motif 18
0
300
600
900
1200
1500
1800

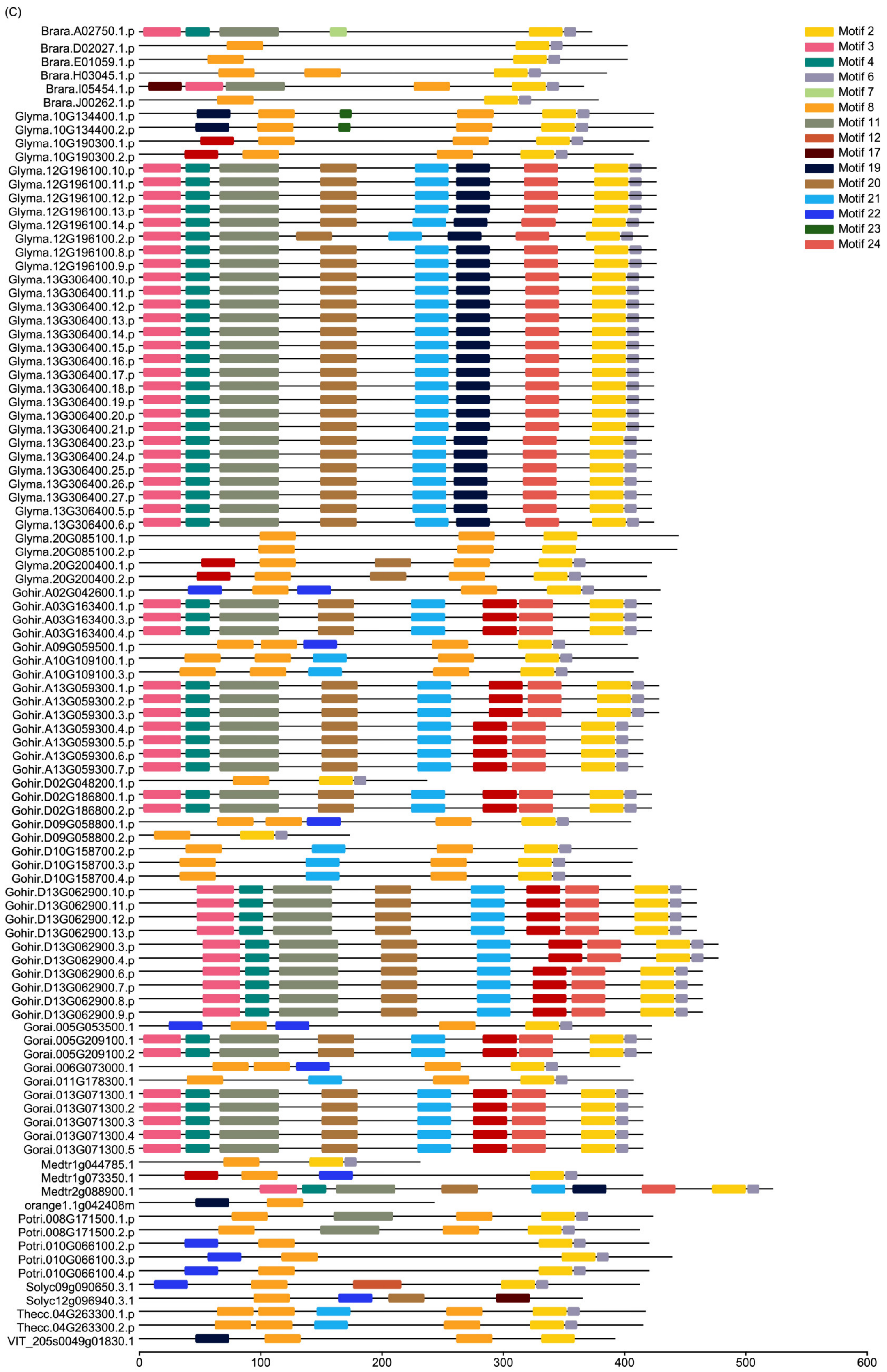

Figure. S2 Motifs of the CCT proteins (A) Motifs of the PRR proteins subfamily (B) Motifs of the COL proteins subfamily (C) Motifs of the CMF proteins subfamily.

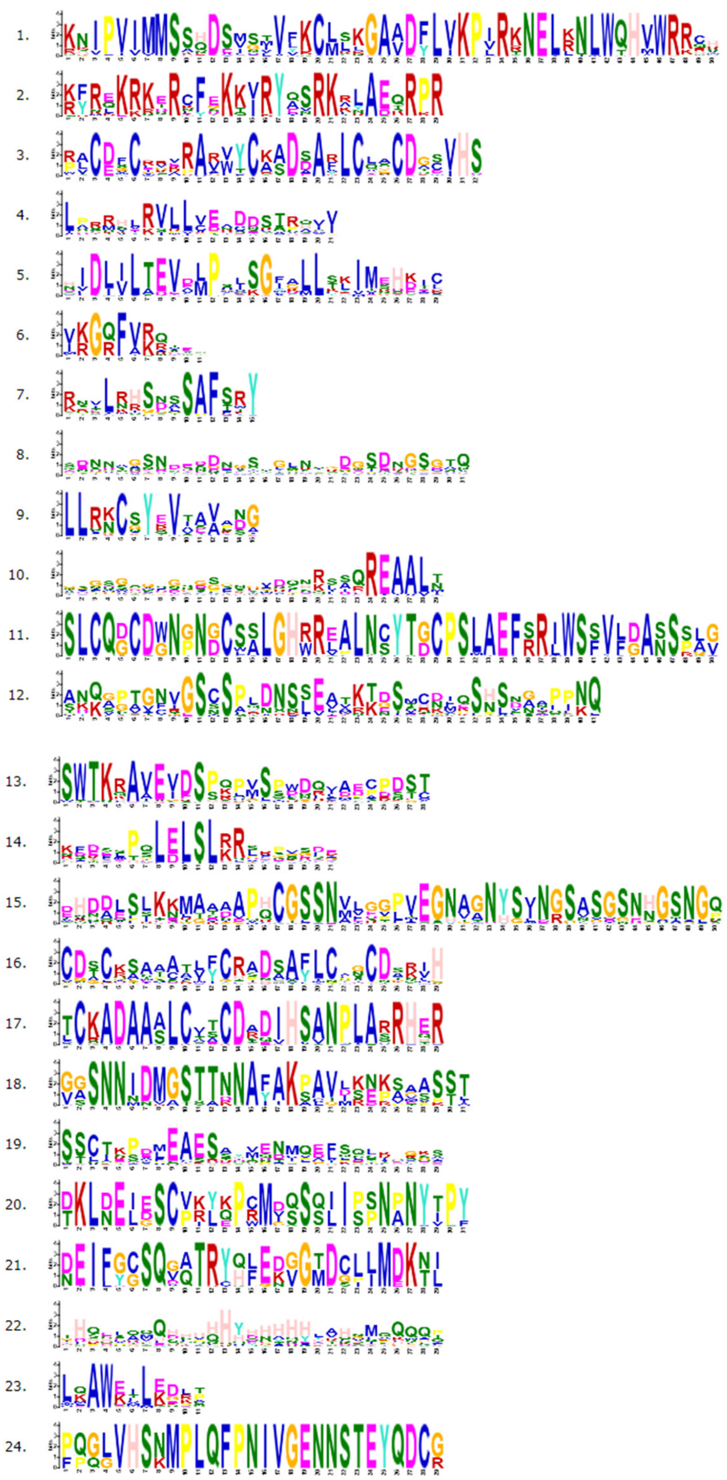

Figure S3 Motifs of the proteins.
